# Interventions and methods to prepare, educate or familiarise children and young people for radiological procedures: a scoping review

**DOI:** 10.1186/s13244-022-01278-5

**Published:** 2022-09-05

**Authors:** Lucy Bray, Lisa Booth, Victoria Gray, Michelle Maden, Jill Thompson, Holly Saron

**Affiliations:** 1 Faculty of Health, Social Care and Medicine, Child Health Literacy, Edge Hill University, Ormskirk, UK; 2 Institute of Health and Wellbeing, University of Cumbria, Cumbria, UK; 3 Alder Hey Children’s NHS Foundation Trust, Liverpool, UK; 4 University of Liverpool, Liverpool, UK; 5 Division of Nursing and Midwifery, Health Sciences School, The University of Sheffield, Sheffield, UK

**Keywords:** Children, Preparation, Radiological procedures, Scoping review

## Abstract

Children attending hospital for radiological procedures can experience uncertainty, anxiety and distress; this can result in sub-optimal experiences for children, poor scan quality and the need for radiological procedures to be rescheduled or sedation to be used. The preparation and education of children before clinical procedures has been shown to have a positive influence on procedural outcomes. This scoping review aimed to locate and examine the evidence relating to non-invasive interventions and methods to prepare, educate and familiarise children for radiological procedures within a healthcare setting. A comprehensive search strategy identified 36 articles. A narrative synthesis approach was adopted to make sense of the key findings. Studies investigated a range of radiological procedures (MRI, plain radiographs, CT, fluoroscopy and Micturating cystourethrogram) using a wide range of interventions (smartphone applications, storybooks, videos, mock scanners) which varied by method, mode of delivery and target audience. The outcomes used to evaluate the value and impact of the interventions are wide, varied and inconsistently applied making it difficult to judge which interventions offer the optimal impact on scan quality, scan completion and children’s experiences. This review highlights that there is a need to further understand which specific elements of the non-invasive interventions ‘work best’ for children. There is a need for consistency on the outcomes measured and for these measures to include child-centred outcomes alongside scan quality and length of radiological procedure.

**Supplementary Information:**

The online version contains supplementary material available at 10.1186/s13244-022-01278-5.

## Key points

- Many non-invasive interventions exist to prepare and educate children for radiological procedures.
- These non-invasive interventions differ in their aims, content, delivery and measures of outcomes.
- These differences make it hard to judge which non-invasive interventions work best.
- A core set of outcomes is needed to enable comparison between different interventions.

## Background

Children frequently undergo a range of diagnostic radiological procedures including plain radiographs, ultrasound (US), magnetic resonance imaging (MRI) and computed tomography (CT). Simple radiological procedures such as plain radiographs are often the first encounter children have with health services with approximately 2 million plain radiographs being conducted on children under 14 years in 2019/2020 [1]. Children and young people undergo over 150,000 MRI scans and 50,000 CT scans each year [1]. Many of these radiological procedures are conducted within adult departments as opposed to dedicated children’s hospitals [2]. Children can often feel anxious, worried and uncertain when they attend hospital for a radiological procedure, due to the unfamiliar environment, noises, sounds and having to keep very still for a good quality image [3]. There is increasing evidence that children having radiological procedures have an improved experience during the procedure and better short- and long-term outcomes if they are prepared and informed about the procedure they are due to have [3–5] and are supported and distracted throughout [3]. Despite significant interest and investment in the development of different mediums and forms of preparatory and educational information, the use and provision of preparatory interventions can be ad hoc and there is a lack of evidence of which methods of delivery work best for children and have the best outcomes [6].

Studies developing and evaluating interventions to prepare, educate and familiarise patients before procedures and health interactions are frequently discussed within the frame of health literacy [7, 8]. Health literacy is gaining increasing traction as a lens through which to understand the individual as well as familial and contextual factors which can influence how a person accesses information, gains knowledge and applies that knowledge to influence their health and healthcare [7]. The concept of health literacy has been used to understand the education and decision-making of parents of children undergoing radiological procedures [9, 10] and of adult patients undergoing radiological scans [11] but has not been used as a framework to examine interventions to prepare, educate and familiarise children prior to undergoing radiological procedures.

Previous reviews have focussed on children being informed and prepared for surgery [12–14] or invasive procedures such as blood tests [15] and systematic reviews to examine methods to distract or support children during procedures [16, 17] but no review has focussed specifically on mapping the different types of interventions and outcomes used in studies examining children’s preparation and education prior to radiological procedures. Therefore, this scoping review aimed to examine the evidence of non-invasive interventions and methods to prepare, educate and familiarise children and young people for radiological procedures within a healthcare setting.

## Method

A scoping review approach was chosen as our focus aligned with Arksey and O’Malley’s [18] review purpose to examine the scope, scale and nature of the current evidence base for preparing, educating and familiarising children and young people for radiological procedures. We used a scoping review methodological framework to guide the review process within the following five sections which will form the structure of the paper; identifying the research aim/question, identifying relevant studies, study selection, charting the data and collating, summarising, and reporting the results [18]. While we acknowledge that the quality appraisal of included papers is not a necessary part of a scoping review [18], the team felt that assessing the quality of the studies was important to inform the nature of the evidence. Ethics approval was not required for this scoping review.

### Review aim

The aim of this scoping review was to examine the evidence of non-invasive interventions and methods to prepare, educate and familiarise children and young people for radiological procedures within a healthcare setting. The objectives were to: outline which interventions are being used, how these interventions are being used and evaluated, which radiological procedures and groups of children these interventions are being used with and the perceived impact of the interventions and methods.

### Identifying relevant studies

#### Search strategy

The literature search to identify relevant studies was conducted in the databases MEDLINE, Cumulative Index to Nursing and Allied Health Literature (CINAHL), the Cochrane Library (CENTRAL), Web of Science (all databases) and PsycINFO. An experienced information specialist (M.M.) conducted the searches. We also scanned the references of the included studies. The search strategy was structured according to the Population, Concept and Context (PCC) approach [19]; the population of children and young people, the context of diagnostic planned radiological procedures and the concept of interventions to prepare, educate or familiarise children *prior* to their procedure. The search strategies were designed using a combination of both subject headings and free text terms and were limited to English language. Full search strategies can be found in Additional file 1: Appendixes A and B.

#### Eligibility criteria

The inclusion and exclusion criteria are outlined in Table 1. This scoping review focussed on the preparation, education or familiarisation of children and young people aged between 5 and 16 years for planned radiological procedures. The rationale for the chosen age range was related to being school-aged children, this aimed to help boundary the search to children who were likely to have reached a level of understanding and cognitive ability. Only peer-reviewed articles, written in English, were included. The search included all sources of evidence from inception to February 2021.

**Table 1:** Inclusion and Exclusion criteria

	Inclusion criteria	Exclusion criteria
Participant	Children and young people aged 5–16 years	Studies conducted in an adult population or where it was not possible to separate out the child data (aged 5–16 years)
Context	Children and young people undergoing a planned diagnostic radiological procedure	Children and young people undergoing urgent or emergency radiological procedures Children undergoing radiotherapy
Concept	Any intervention/method (of any type) designed to directly prepare/familiarise or educate children prior to their radiological procedure Interventions/methods used or that occur prior to the procedures	Interventions focusing on the education or training of healthcare staff Interventions used solely during the procedure, e.g. distraction techniques
Publication	Empirical research studies evaluating the effect, impact, value or influence of interventions/methods English language only	Studies that only describe the intervention’s development or use within practice, with no evidence of evaluation Editorials, opinion pieces

### Study selection

The screening and review process was facilitated by using Covidence [20] throughout. The first two steps of the selection process were the title and abstract screening and subsequent full text screening. Two reviewers (L.Br., H.S.) independently screened the studies during both stages of the screening process. Any disagreements were flagged within the Covidence platform and these were reviewed and discussed between the reviewers until consensus was reached.

### Charting the data

Data extraction or charting was conducted and mapped onto a form structured to capture details of the empirical study (e.g. author, date, country, study design), characteristics of the intervention, delivery of the intervention/method and the outcomes of the study (Table 2, a more detailed chart is included as an Additional file 1). Five reviewers (L.Br., H.S., V.G., L.Bo., J.T.) conducted and checked the charting of data for each included paper.

**Table 2:** Data extraction of the included studies

Author/Year/ Country	Aim	Study Design	Participants age & condition/s	Intervention characteristics	Intervention delivery	Data collection methods	Outcomes	Results/Findings
Ashmore et al. (2019) [34] UK	To gain feedback on the initial implementation of the app to help inform further enhancements of the resource	Descriptive quantitative design	23 children (median age 9 years, range 4 to 12 years) who had never had an awake MRI (n = 19/23) or had had an MRI more than 1 year ago (n = 4/23)	An app (targeted at 4–12 year old children) to produce an immersive 360° VR experience of the entire MRI journey	The resource was developed to be used by health play specialists in hospital or at home where a disposable Google Cardboard version 2 headset was mailed to patients	Locally developed parent questionnaire Locally developed HCP questionnaire	Ease of use Helpfulness of information, Enjoyability of the resource	23 parents/carers answered the questionnaires The parent questionnaire highlighted they felt that the resource had a positive impact on their child The feedback showed a positive impact of the app on parents, the app allowed them to better understand their child’s upcoming MRI, helping to reduce their own anxieties and enabling them to better prepare their child 10 health professionals answered the questionnaire and reported that the preparation resource was a useful tool Of the 5 patients originally booked for MRI under GA, 4 were able to tolerate an awake MRI
Barnea-Goraly et al. (2014) [35] USA	To judge the feasibility of using a behavioural desensitisation program to yield high quality brain MRI scans in sedation-free children	Descriptive quantitative study	222 children (4–9.9 years), 147 with type 1 diabetes and 75 age-matched non-diabetic controls	Multi-media resource and mock scanner to prepare and desensitise children prior to an MRI including preparation at home and on arrival at the hospital	One part delivered by parents at home and one part delivered by staff within the radiology centre	Each scan taken was reviewed independently by 2 staff to discern if they were useable and of good quality	Useable scan First attempt successful scan	Brief behavioural training can lead to a high rate of success for obtaining excellent-quality brain MR images without sedation from very young children
Bharti et al. (2016) [24] India	To evaluate the effectiveness of an MRI specific play therapy intervention on the need for sedation in young children	Randomised control design	79 children (40 intervention group, 39 control group) undergoing MRI for neurological and non-neurological conditions. Children’s mean age was 7.11 years. Children with previous experience or cognitive disabilities were excluded	Children in the intervention group received MRI customised play therapy with a doll-sized mock scanner on the day of the MRI investigation	Play therapy sessions were conducted by a paediatrician and a trained medical social worker on the day of the MRI investigation	The scan quality was rated on a five-point scale by an experienced radiologist If the child did not cooperate with the procedure within 20 min the standard protocol for sedation was used	Number of children requiring sedation Quality of the scan achieved	The study demonstrated the effectiveness of MRI customised play therapy with children prior to the scan as it significantly reduced the need for sedation and anaesthesia in a significantly greater proportion of children as compared to the control group
Capurso et al. (2020) [36] Italy	To establish and evaluate an MRI preparation procedure	Retrospective cohort study	66 children (3–14 years; mean 7.52 y, SD 2.55 y, 63% male) were prepared to undergo MRI scans	Play-based stimulation MRI training using a 8-step protocol including a booklet, hearing the MRI sounds and role-play activities	The training protocol is completed by two volunteers An average intervention lasted approximately 70 min	Quality was measured through a 4 point motion artefact scale at 3 points during the MRI	MRI pass rate Quality of the obtained images	All of the children succeeded in completing the preparation. Out of the 66 prepared children, 62 (93.9%) completed the MRI scan Out of 66 children who underwent the MRI preparation, 61 (92.4%) achieved clinically diagnostic scans
de Bie et al. (2010) [37] The Netherlands	To evaluate the use of a mock scanner training protocol for preparation of children of 3 to 14 years of age for both structural and functional MRI	Descriptive quantitative design	90 children (median age 6.5 years, range 3.7–14.5 years) 47 children (MRI group) 43 children who were recruited for a controlled study on brain development, intelligence, and cognitive outcome	Full-size mock scanner training, including verbal instruction, the various MRI sounds, role playing activities and practicing lying still	A paediatrician or experienced child-life specialist conducted the training session A training session lasted 30–60 min Delivered before the MRI	The quality of structural MRI scans was rated by a five-point rating scale by an experienced radiologist Success rate of structural scan sessions was defined as the proportion of children with structural MRI scans with score 1–3	Pass rate of the mock scanner training sessions (ability to be still for 5 min) MRI scan quality	The overall pass rate of the mock scanner training sessions was 85/90. Structural scans of diagnostic quality were obtained in 81/90 children, and fMRI scans with sufficient quality for further analysis were obtained in 30/43 of the children
Carter et al. (2010) [55] Australia	The aim was to determine whether the introduction of a mock MRI service assisted in reducing the number of GAs being performed on children undergoing MRI	Retrospective audit	Children aged 3- 14 years 11 months who completed an MRI 4 groups of children accessed different preparation elements	Graded exposure to the MRI process and to practice for the MRI in a 1 h pre-booked session including instruction, sounds of the MRI, role-playing and practicing lying still	Paediatric occupational therapist One hour session	Retrospective audit of the picture archiving communication system (PACS), medical charts and anaesthesia records	The need for GA Completion of the mock MRI Number of MRI scans performed Quality of the MRI scan	In the pre-mock period 756 children underwent 1,072 MRIs with a GA rate of 26.8%. In the post-mock period 875 children underwent 1,205 scans with a GA rate of 18.2%. This overall difference of 8.6% was calculated as being *statistically significant*
Cavarocchi et al. (2019) [38] Italy	To evaluate the introduction of the Kitten Scanner training protocol on children undergoing an MRI	Retrospective cohort study	Children aged 4–14 years (n = 570) who underwent normal preparation and (n = 891) who underwent the MRI examination after introduction of the Kitten scanner	Play therapy training sessions. Children were engaged in a simulation of the real MRI investigation with a toy-model scanner called Kitten Scanner	Child life specialist Delivered the same day of the MRI in a quiet room in the department The duration session could last between 30 and 40 min	The quality of MRI images taken after the Kitten Scanner training was evaluated by an experienced radiologist	Number of children undergoing a brain MRI scan without sedation Quality of scans	After the introduction of the Kitten Scanner training, there was a significant increase in the number of children undergoing the brain MRI scan without sedation, both for the total group (*p* < .001) as well as for the 4 to 9 years of age group (*p* < .001) Children who received most benefit from this training were in the 4 to 9 years of age group All brain MRI examinations performed without sedation after the Kitten Scanner training were of sufficient quality to be used for diagnostic purposes
Cedja et al. (2012) [56] USA	To examine the use of the Preparation and Support Procedures (PSP) program and its effect on the ability of young children to successfully complete brain MRI or liver R2*MRI exams	Retrospective review of medical records	71 children with sickle cell disease (SCD) aged 5.6–12.9 years (median age 9.9 years) who underwent a conventional MRI of the brain or an R2*MRI of the liver	The play therapy session used a small model MRI machine, pictures of the MRI suite and recordings of MRI sounds to prepare the child for the procedure	Child-life therapist	The quality of images was evaluated by a neuroradiologist or paediatric radiologist	Quality of the scan Use of sedation or anaesthesia	The child life specialist offered PSP to 33 (46.5%) children; Children receiving PSP had 8.5 (95% CI 1.7, 43.3) times the odds of successfully completing an interpretable MRI exam compared to those who did not receive PSP (P = 0.0098). Of the 30 children who successfully underwent MRIs with the PSP intervention, 20 (67%) had required sedation/anaesthesia for a previous MRI
de Amorim e Silva et al. (2006) [57] Australia	To evaluate the effectiveness of a practice magnetic resonance unit in preparing children to undergo an MRI	Retrospective review of medical notes	134 children undertook a practice MRI (aged 4.1–16.1 years, median age 7.7 years, 47% boys)	Practice full-scale mock MRI children are shown a storybook of a child having an actual MRI with photographs and practice lying still	Most practice MR sessions take 30 min to an hour	Retrospective review of the records to assess whether the child had passed or failed the practice MRI intervention. Review of the scan quality	Ability to have an MRI without a GA Scan quality	In all, 120/134 (90%) passed the practice session; 117/120 (98%) of those subsequently had a clinical non-GA MRI and 110/117 (94%) passed
Durand et al. (2015) [45] USA 45	The aim of this study was to assess the impact of child life evaluation for children undergoing MRI before referral for general anaesthesia	Before and after design	Children aged 5- 18 years without severe neurodevelopmental delay Baseline (before) group (n = 47 children) Intervention group (n = 263 children)	Child life specialist preparation, information giving and coping strategies training	Child life specialist Day of the MRI scan	Data collected included whether the scan was successfully completed; and whether the scan was performed under general anaesthesia, with diazepam, or with no sedation	Successful completion of the scan Need for GA Need for sedation	The difference in the need for general anaesthesia between the time periods was highly statistically significant (*p* < .001) During the baseline period, 47 patients were referred for child life evaluation, all of whom eventually underwent successful scans. During the intervention period, 263 patients were referred for child life evaluation. the scan success rate in this population was 98.4%, with 2 failures due to anxiety
Fegley (1988) [25]	The purpose of this study was to examine the effects of choice in pre-procedure instruction on: a) children’s search for information behavioural responses and self-reported distress	Randomised controlled trial	61 children ranging in age from 4 to 12 years (M = 7.45, SD = 2.62 who were scheduled for a routine intravenous pyelograms (IVP) and/or voiding cystourethrograms (VCUG)	The child was randomly assigned to one of the following groups *Contingent Instruction.* Individualised education based on children’s questions and information needs *Noncontingent Instruction* Predetermined standard information about the radiologic procedure	Nurse Delivered on the day of the radiological procedure	Observations of the scan at three time periods during the procedure focussed on children’s information seeking, the Manifest Upset Scale, the Cooperation Scale and the self-report of distress		The type of instruction was significantly related to the search for information Older children spent more time searching for information, (pr = 0.28) were more cooperative laying on the table (pr = 0.50) and during the intrusive procedure (pr = 0.45) displayed less upset behaviour getting on the table (pr = 0.40) and reported less distress (pr = -0.40)
Fraser (2019) [39] USA	To examine the effects of choice of information in pre-procedural instruction on children's responses to select radiologic procedures	Electronic medical record review	958 children aged 3 and over have participated in the programme over a 6-year period	Patient Awake While Scanned (PAWS) preparation and support program which involved phone assessment 2 weeks before MRI, images of the scanner, pre-scan CCLS meeting to provide individualised support and coaching, explanation, and support from the CCLS, MRI technologist, and caregiver during the MRI	Mostly Certified Child Life Specialists (CCLS), but also MRI technologist and caregiver	Not stated	Completion rate Cost savings	A 96% rate of successful scan completion without sedation This program has minimised health risks associated with anaesthesia use in MRI and lowered the overall cost to families and the institution. There is a cost saving of $241.82 an hour in salaries alone
Gebarski et al. 2013 [26] USA	To assess the efficacy of a cartoon and photograph montage storybook in preparing children for VCUG	Randomised prospective study	100 children (87 girls, 13 boys) Mean age 5.3 years 50 children received the storybook and 50 did not	A storybook with cartoon characters superimposed on photographic backgrounds of the radiology department and fluoroscopy suite. An accompanying stuffed animal was provided to enhance the parent–child interaction during reading	Delivered by the parent/carer at home	Parent questionnaire completed after the VCUG to rate their child’s tolerance, use of the book and other sources of information used VCUG technologist (blinded) rated each child’s tolerance/distress on a scale modified from the Groningen distress scale at 2 points in the procedure	Child distress	The association between experiencing the storybook and high performance scores as rated by the technologist was statistically significant (*p* value = 0.0092). Children prepared with the storybook were 2.7 times as likely to score high
Hallowell et al. (2008) [54] Australia	To determine the effectiveness of a PMRI service in helping children cope with diagnostic MRI and to reduce the requirement for GA	Clinical prospective audit	291 children (aged 3 years 7 months to 17 years, mean 7.9 years) undergoing an MRI	Play MRI process including photo story book, discussion of the steps and sensations involved, tour of the PMRI unit, choice over distraction technique and practicing lying still	Educational play therapist Session delivered on the day of the MRI	MRI scan results were reviewed by a paediatric radiologist to ascertain scan quality	MRI scan quality	Of the 291 children who underwent a PMRI, 218 (74.9%) passed, and 227 (78%) went on to clinical MRI without GA. Of these 227 children, 198 (87.2%) had passed a practice MRI, 1 (0.4%) had failed and 28 (12.3%) had been considered borderline. A diagnostic study was achieved in 218 (96%) of the 227 children who underwent a clinical MRI without GA
Han et al. (2019) [27] Republic of Korea	To evaluate whether virtual reality education for paediatric patients before chest radiography could reduce anxiety and distress in children and improve the radiographic process	Randomised clinical trial	99 children aged 4 to 8 years who underwent chest radiography	Virtual Reality group 3-min virtual reality education explaining chest radiography. Delivered 5 min before the procedure Control group simple verbal instruction	The VR group received a 3-min VR educational presentation regarding the radiologic process with a head-mounted VR display 5 min before entering the radiography room	*Children’s stress and anxiety* Amended version of an OSBD scale *Parents’* Self-reported satisfaction *Procedural characteristics* Procedure time, number of repeated procedures, difficulty of the chest radiographic imaging	Child anxiety and distress Need for parental presence Parental satisfaction score Procedure time Number of repeated images Process difficulty score	The number of less distressed children (OSBD score, < 5) was significantly higher in the VR group (38 [77.6%]) than in the control group (26 [52.0%]) and the degree of stress and anxiety measured was significantly lower in the VR group than in the control group. The mean (SD) score for parental satisfaction (9.4 [1.4] vs 8.6 [2.0]) was higher in the virtual reality group than in the control group
Hartman et al. (2009) [28] USA	The purpose of this study was to assess if pre-procedural education decreased pre-procedural stress and anxiety for children undergoing MRI	Randomised controlled trial	50 children (7–12 years old, without intellectual disability) undergoing an MRI 25 in control group and 25 in education group	*Education group* 24- page photo diary provided for children to read describing what children can expect (sounds, sensations)	Paper implies the photo booklet was read by families	Data were collected at three points in time, enrolment, before MRI, after looking at the intervention Children completed the Children’s Stress Symptom Scale and the Revised Children’s Manifest Anxiety Scale (RCMAS) Parents completed a survey on the perception of their child’s readiness for MRI (VAS) & parental satisfaction with the education provided to their child (VAS)	Child anxiety Child stress Parental anxiety	The results of this randomised controlled study suggest that a photo diary does not reduce pre-MRI stress and anxiety in school-aged children and does not improve satisfaction with education in parents who accompanied children undergoing an MRI scan
Hogan et al. (2018) [29] USA	To evaluate the effectiveness of an educational video vs. standard of care in improving relaxation and procedural understanding among paediatric patients undergoing a magnetic resonance imaging (MRI) procedure	Pilot randomised controlled trial	50 children 6 to 17 years of age undergoing an MRI Half of the children had undergone a MRI previously and nearly half required an intravenous catheter for contrast dye administration	*Educational group* 7 min MRI educational video on a portable electronic device in the MRI waiting area including information on what a MRI is and how images are taken, the MRI noises and the healthcare team they are likely to meet during the course of their visit	Self-administered video in the MRI department	Children > 7 years were asked to circle their level of relaxation using a 10-point VAS before their scan After the scan children rated how well they understood what they were told about the MRI (VAS) and open ended questions asking what children found most helpful about the MRI education	Child self-reported relaxation Child self-reported knowledge	With regards to patient understanding of the MRI procedure, patients in the intervention group had higher levels of mean understanding scores than those in the standard care group. The educational video was associated with increased relaxation among children, with the indication that it may be the most effective among older, adolescent children A total of 26 patients, half from the control group and half from the intervention group responded that the educational video was helpful in increasing their awareness and understanding of the MRI process
Johnson et al. (2009) [46] USA	To evaluate whether an instructional colouring book used by a parent along with the child would reduce anxiety among paediatric patients about to undergo a radiology imaging test	Before (control) and after (intervention) trial	3- to 10-year-old children (mean age 6.1 years) who were scheduled for outpatient CT, fluoroscopic, ultrasound, or nuclear medicine Excluded MRI and brain imaging	An instructional colouring book, ‘Radiology for Kids: Take a Tour with Garfield’ included cartoon depictions of equipment and brief explanations of radiology imaging tests as explained by the Garfield character and Odie undergoes the tests	The radiology colouring book was given to parents and patients for review while in the waiting room before their radiology tests Parents and self-directed educational	*Parents* Parental anxiety—Modified Amsterdam Preoperative Anxiety and Information Scale (APAIS) A VAS to measure parental estimation of patient anxiety levels just before the imaging test Four specific Likert-scale questions related to the utility of the colouring book *Children* Modified Faces Pain Scale-Revised (FPS-R) to estimate patient anxiety	Parent reported child anxiety Child anxiety	Neither parental estimation of patient anxiety (from the VAS) nor patient anxiety score (modified FPS-R from the patient) differed significantly between the control group with no colouring book and the intervention group who reviewed the colouring book The parents and children reported that the colouring book helped them better understand the radiology imaging test and made them less worried about the test my child had
Johnson et al. (2014) [30] USA	To examine effectiveness of the social script intervention “Going to Imaging” application (app) on anxiety, challenging behaviours, and procedure duration among children with ASD, and the anxiety of their parents	Randomised controlled trial feasibility study	32 parents and 32 children (age 0–19 years) in the study with a mean age of 10.3 years (SD = 5.1) Children had an ASD diagnosis by parent report Children with planned sedation or anaesthesia were excluded	Four procedure specific apps for MRI, CAT scan, X- ray and nuclear medicine. Each app has 10 screens of photos. The script was based on social script formatting that prepares a child by breaking down a procedure into steps and provides a script of responses	The experience of the child using the app was estimated to be 5 min A researcher delivered the intervention	The study involved data collection immediately before and after the iPad app intervention and during imaging Parents rated their anxiety on the State-Trait Anxiety Inventory for Adults (STAI-S) Child stress was measured by HR and BP monitor Child behaviour was measured with the behavioural observation tool for children with ASD in the healthcare setting (BOT)	Stress response Observable child challenging behaviours Procedure duration	Pre and post intervention change in mean child HR and systolic BP was greater for the intervention group compared to the control group Children in the control group had higher mean number of challenging behaviours The imaging procedure's time in the imaging room was less for the intervention group compared to the control group Change in parents’ state anxiety was greater for the interventional group compared to the control group
Karakas et al. (2015) [40] Turkey	To demonstrate whether pre-scan training and orientation affect fMRI compliance of children with ADHD and determine whether this compliance is modified by state anxiety	Part of a large-scale descriptive quantitative design	77 boys aged 6–12 years—a subsample (53 boys with ADHD and 24 boys in the control group) of the larger study protocol (70 boys with ADHD and 38 boys in the control group)	Children were taken on a tour of the department, shown the MRI scanner, introduced to staff and technicians and watched another child being scanned. Just before the MRI, children were individually trained and practice trials were repeated until the children understood the task	Study coordinator Preparation and training were conducted on the day of the scan	State anxiety scores	Scan success (acceptable amount of head motion) Repetition rates Cancellations due to refusals Expression of distress while in the scanner	Compliance was not significantly different between ADHD and control groups based on success, failure, and repetition rates of fMRI. Compliance of ADHD patients with extreme levels of anxiety was also not significantly different
Mastro et al. (2019) [58]	To evaluate the effectiveness of an anaesthesia-free patient- and family-centred intervention through an analysis of MRI quality, health-care costs, and operational efficiency as compared with other approaches	Retrospectivereview of electronic medical records	500 children aged 3–17 years, who underwent outpatient MRI 125 children in each of four different intervention arms	Pre MRI preparation session included a preparation book on iPad (with sounds, pictures, and text) covering all stages of the MRI visit. A medical play session led by the child with a mock toy MRI scanner with figures and dolls. Practice of coping techniques such as keeping still, guided imagery, audio music, and movie with MRI goggles	Nurse developed CCLS supported	MRI quality on a 5 point likert scale Hospital charges Procedural time	Image Quality Hospital Cost Procedural Time	The PFC/NA intervention group was found to have statistically significant lower and shorter procedure times and 96.8% of the MRI images were of acceptable or better quality than those of the SC/A and CCLS/A groups
McGlashan et al. (2017) [4] UK	To examine whether the animated educational video provides an internet-based tool for MRI preparation	Prospective cohort study	6.5 to 11.5 years 9 children with A-T (neurodisability with movement disorders) and 12 undergoing a clinical research MRI scan	An internet-based educational 3 min animated video The animation used was an updated version from the Szeszak et al. (2016) study	Self-directed Participants were sent an internet link to the animation prior to the MRI scan appointment	Locally developed questionnaire with closed responses (Likert and yes/no) and some qualitative responses *Children* Frequency of watching video and perceptions of the video Pre-scan perceptions (worry, expectations) Post-scan perceptions (whether the animation helped them undergo the scan, whether it helped them feel less nervous) *Parents* Pre-scan questionnaire on whether the animation was viewed, perceived positively by their child, helped prepare their child for the scan	Understanding of MRI scan Likeability of the animation Usefulness of the animation in preparing the child for the MRI	The children rated that they liked the animation and had a good pre-scan understanding of the MRI. The impact the animation had on preparing the children for the MRI was rated good The results indicated the animation had a larger impact on younger children. Nine children across both groups commented they wanted more realistic and louder noises in the animation and six children wanted a better indication of scanner size Results from the parent/guardian questionnaire showed 100% of parents agreeing that the animated film helped prepare their child for the MRI scan 19 of 21 children completed the core MRI research protocol
Morel (2020) [47] France	Evaluated the impact of a teddy bear-scale model of a mock MRI scanner on the anxiety experienced by parents and their children during MRI without general anaesthesia	Prospective controlled trial	91 children (46 girls, 45 boys), aged 4 to 16 years who presented to the ambulatory tertiary centre for an MRI scan Children were excluded because of severe cerebral palsy, severe attention deficit hyperactivity disorder or a lack of communication skills	Mock scanner specially designed to look like a toy to the scale of a teddy bear	MRI technologist Duration not stated	*Ambiance of the preparation* *room rated on a* 4-point Likert scale *Child Anxiety levels were rated on a* VAS at three time points, in the waiting room, after the preparation and after the exam Overall appreciation of the MRI examination was collected at the end of the procedure	Ambiance of preparation room Child anxiety level	Anxiety levels before the MRI examination were lower in children after the installation of the teddy bear-scale model of an MR scanner The anxiety level estimated by children was significantly lower after the explanations in the post-mock period. a significant difference between anxiety score in the waiting room and after the exam was also observed Children and parents gave free comments: They reported that they understood the MRI device much better
Nordahl (2016) [48] USA	To develop improved and safer methods for obtaining high-quality images in a broader spectrum of children with ASD	Cohort study	17 children aged 9 to 13 yearswith ASD and intellectual impairment	*Pre-visit preparation (*Structured interview, Video Model, mock scanner room, 3 T MRI suite) *Mock MRI session* Full-size mock scanner practice; lying down, tolerating movement of bed into scanner, tolerating noises, staying still)	Behaviour analyst, parents, and the research team	Quality assurance procedure to meet the QA threshold	Scan success rate Scan quality	The success rate in acquiring T1-weighted images that met quality assurance for acceptable motion artifact was 100%. The success rate for acquiring high-quality diffusion-weighted images was 94% The number of mock training sessions never exceeded into two visits. All four participants with IQs in the normal range required only one mock visit
Ong et al. (2018) [31] Singapore	To assess the effectiveness of pre-scan videos on children having an MRI examination	Prospective randomised controlled trial	789 children (mean age 11.6 years) The children were randomly assigned into 3 groups (control, regular cartoon video and interactive video combined with regular cartoon video groups)	A 2-min regular cartoon of a potato character undergoing an MRI examination, and an interactive video where a child is able to assist a panda character undergoing an MRI examination with MRI sound included	Children were shown the videos in a separate waiting area prior to their MRI	Children were surveyed before and after the videos to assess the self-reported duration that the child believes he/she can lie still for the MRI examination	Need to anaesthetise or repeat the MRI sequence	Viewing of videos did not have a significant effect on GA requirement even after adjusting for confounding effects of age, gender and prior MRI experience The results of this prospective randomised controlled trial suggest that children benefit from the pre-MRI videos, as evidenced by the significant reduction in the requirement for repeated MRI sequences due to motion artefacts and improvement in the confidence of children in staying still for at least 30 min
Pressdee et al. (1997) [59]	To describe the implementation of a play preparation programme	Retrospective description	169 children aged 4–8 undergoing an MRI plus any older children who were perceived as benefitting from preparation	Play therapy and colouring book The play specialist explains the procedure to the child and parents. Photographs of children or a teddy bear undergoing MRI. A small model of the MR unit, a tape recording of the noise produced during the investigation	Play Specialist	Not stated	Completion of scan	Only 1/169 of the children required MRI under GA Parents felt that this preparation had been of considerable benefit in decreasing stress and anxiety caused by the examination
Pua et al. (2020) [49]	To familiarise children to MRI scanner environment and improve tolerance to loud and repetitive scanner noise	Descriptive quantitative study	12 children aged 5–18 (monozygotic twins concordant or discordant for ASD)	Parents took part in a brief clinical interview with a psychologist and provided with an MRI familiarisation package (MRI orientation video, introducing child to locations in hospital and MRI scanner, Mobile app with interactive games, on-site visit – mock MRI training session)	Psychologist interview Parent delivered video and app	Measurements from an accelerometer device MRI quality indices	Scan duration Scan completion	Only one participant failed to meet criteria for acceptable levels of head motion and image artefact control
Rothman et al. (2016) [32] Isreal	To evaluate a program that prepares children for MRI, by means of full or partial instruction	Prospective randomised study	64 children full instruction aged 8 years ± 2 57 children in partial instruction aged 8 years ± 3	64 children received full interactive instruction that included an instructional booklet, movie and simulator practice 57 children received partial instruction that consisted of only the booklet Instruction occurred while the child waited for the scan	Health professional	Spielberger state anxiety inventory. Parents were asked to rank 10 questions that referred to current feelings	Anxiety Need for anaesthesia	The frequency of anaesthesia was statistically significantly lower in children who received full as compared to partial instruction The median anxiety level prior to instruction was higher than the median level after instruction for both the partial and full instruction groups
Szeszak et al. (2016) [50] UK	To evaluate an animation in preparing children for an MRI scan	Descriptive quantitative design	23 children (mean age of 7.65) Children with previous experience of MRI scans, history of neurodevelopmental disorder or poor English language comprehension were excluded	The animation lasted 3 min and follows Jess as she experiences an MRI scan. The design of each scene in the animation was based on real-life MRI equipment at the particular department	Self-directed	Children rated their knowledge of MRI and anticipated anxiety on a Likert scale *An interview e*xplored children’s understanding, anxiety and opinions of the animation	Knowledge Child anticipated anxiety Opinions about the animation (usability and retained attention)	There were statistically significant improvements in children’s knowledge in 3 of the 7 knowledge questions# Questions regarding anticipated anxiety relating to MRI showed significant improvements of + 1 in median score 100% of participants responded that they liked the way the animation looked, that the people in the animation looked friendly, and that they found it easy to hear what the people were saying. 95.7% of participants reported that they liked the MRI animation overall. 87% of participants reported that they would like to see more animations of this sort for other hospital tests and treatments
Thung (2018) [51]	To determine whether the Yale Preoperative Anxiety Scale (mYPAS) obtained before MRI simulation can effectively predict success of MRI without	Before and after cohort design	80 participants (43 boys and 37 girls). Mean age of 8.5 SD 3 years	Simulation based training using a practice MRI scanner Practice MRI scanner	Child life specialist	Scan duration Child anxiety assessed using mYPAS	Need for sedation or anaesthesia for MRI Child anxiety	69 from 80 did not require anaesthesia for MRI after simulation Overall study cohort mYPAS scores improved from 31 (± 11) to 27 (± 9) 11 children were unable to complete scan due to nervousness or anxiety and inability to lay still
Tornqvist et al. (2015) [42] Sweden	To determine whether children who receive age-adjusted routines can undergo MRI without deep sedation/anaesthesia	Cohort design with two groups studied at different time period	Control group (n = 36 children) and intervention group (n = 33 children) who attended scheduled MRI scans for head or head and spine examinations	All children in the intervention group received; a booklet and a storybook sent home, a ‘doll-size’ model of an MRI scanner made with an MP3 player with the MRI sound recorded was shown to the child at the day care unit along with a DVD film while undergoing MRI	Not documented	Data collection included procedural information (sedation/anaesthesia, length of the scan, successful completion), image quality and motion and the parents recorded their satisfaction with the care of their child (Healthcare Satisfaction Module specific for Hematology/Oncology) and costs for the examination	Number of children who successfully went through MRI without deep sedation or anaesthesia Image quality concerning motion artifacts Parents’ satisfaction with the care Scan costs	In the control group, 30/36 needed sedation/anaesthesia, in the intervention group 3/33 needed sedation/anaesthesia Comparison of parents’ satisfaction showed no significant difference between the groups
Train et al. (2006) [43] UK	The aim of this study was to evaluate a psychological intervention designed to reduce distress in children undergoing 99mTc-DMSA	Retrospective (control group) and prospective (intervention group) cohort study	121 children in total. 81 children in the control group (mean age of 3.8 years (SD 3.2); 40 children in the intervention group (mean age 2.9 years, SD—2.4)	Intervention group families were sent a brightly coloured photo-booklet depicting a child having a scan. There was also a letter giving advice on preparing children for medical procedures and the waiting area was enhanced to be more child-friendly	Researcher	Parental satisfaction (Likert scale) completed after their child’s scan Rates of sedation and procedure failure established from the medical notes Parents completed the Spielberger Anxiety Questionnaire Child’s distress was rated by the doctor (VAS) The image quality was blind rated by a consultant radiologist	Child distress Need for sedation Parental anxiety Image quality	Sedation rates were significantly lower in the intervention group. The rates of failed procedures and use of intravenous sedation were also lower in the Intervention group Satisfaction rates were significantly higher in the intervention group The children’s distress scores before the procedure were lower in the photo-booklet group than in the standard care group but were not significantly different The qualitative comments suggest that the provision of additional information about what families should expect on the day, set out in an appealing child-centred way, increased levels of cooperation and satisfaction
Utama et al. (2019) [33]	To investigate whether the use of an interactive educational animated video is non‐inferior to showing two videos in improving children's cooperativeness during MRI scans	Prospective, randomised, non‐inferiority trial	558 children (aged 3 to 20 years)	Group 1 children (n = 281) watched a 2-min regular animated video of a boy undergoing an MRI scan *and* a 2-min animated interactive video where children help a panda through an MRI scan Group 2 children (n = 277) watched the interactive animated video only	The videos were watched in the waiting area prior to children attending their MRI scan	*Children were asked to assess their confidence in staying still for at least 30 min both before and after watching the videos* *Recorded number of children requiring repeat MRI or GA*	Repeated MRI sequences, Need for general anaesthesia (GA) Improvement in children's confidence of staying still for at least 30 min	In the interactive video group 31% (n = 86) needed repeat MRI, 0.7% needed GA and proportion of children who reported confidence to stay still increased by 22.1% In the combined video group, 36.3% (n = 102) children needed a repeat MRI, 2.1% of children needed a GA and the proportion of children who reported confidence to stay still increased by 23.2%
Waitayawinyu (2016) [52]	To identify the success rate of MRI in 6–15-year-olds, non-sedative paediatric patients after watching MRI introductory video	Prospective interventional study	55 children (aged 6–15 years) Children were excluded if they had neurovascular diseases	An introductory video which was presented as both cartoon animation and real MRI set up, included scanner suite introduction, how the scanner works, patient’s position in scanner and audio of the scanner. Patients would then make decision whether they needed any sedation for the scan session	5 min	Data collection included procedure time, quality of MR imaging and anaesthetic data	Scan quality Scan completion Use of anaesthetic and/or sedation	After watching the introductory video, 37 participants (67.2%) decided to proceed with non-sedative option. Ninety-four percent of non-sedated group (35 participants) went through MRI scan course successfully while two cases were unable to complete the scan and requested sedation afterwards
Williams & Greene (2015) [44] Australia	To examine the impact of the app on children’s anxiety when undergoing medical imaging	Prospective cohort study	50 children in the control group 50 children in the intervention (app) group	An App for radiology procedures which includes three training games and explanatory videos. There is also information for families including tips, things to practice, wearing the right clothes and frequently asked questions	Children can access the app either before coming to hospital or when at hospital through the Play Therapists in the Medical Imaging Department	No information on the data collected	Anxiety Compliance Time taken to be ready for imaging	The average time taken for patients to be ready for imaging reduced. The average compliance issues reduced and the average anxiety rates improved. Additionally, two patients in the group who did not have the app failed to undergo imaging, while all patients who had the app were able to undergo successful imaging
Yamada et al. (2020) [53] Japan	To explore the generalisability of preparation for functional paediatric neuroimaging to clinical simulation in nursing	Retrospective review	241 children aged 4–17 years	A simulation protocol using a mock scanner preparation with sounds immediately before an MRI being performed	Experienced staff The average simulation time was approximately 40 to 60 min Completed just before their scheduled MRI studies	Medical case note review	Scan completion	Studies were successfully completed for 100 (98.0%) participants with TD and for 130 (93.5%) participants with NDDs, resulting in The study suggests, this device can help participants become more relaxed

#### Quality assessment

Even though a scoping review methodological framework does not require quality appraisal, a critical appraisal of the selected papers was conducted using The Mixed Methods Appraisal Tool (MMAT) version 2018 [21]. This tool was chosen as it is validated and appropriate for appraising quantitative, qualitative and mixed methods research [22]. Two reviewers from the team (LBr, HS, VG, LBo & JT) were allocated to each paper to conduct quality appraisal and quality assessments were then cross-checked. The quality assessment of the included studies is detailed in Table 3. No studies were excluded as a result of the quality appraisal process.

**Table 3:** Mixed Method Appraisal Tool quality appraisal for the included studies

	Screening questions		2. Randomised controlled trials				
Paper	S1. Are there clear research questions?	S2. Do the collected data allow to address the research questions?	2.1. Is randomisation appropriately performed?	2.2. Are the groups comparable at baseline?	2.3. Are there complete outcome data?	2.4. Are outcome assessors blinded to the intervention provided?	2.5 Did the participants adhere to the assigned intervention?
Bharti et al. (2016) [24]	Yes	Yes	Yes	Yes	Yes	Yes	Yes
Fegley (1988) [25]	Yes	Yes	Yes	Yes	Yes	Yes	Yes
Gebarski et al. 2013 [26]	Yes	Yes	Yes	Cannot tell	Cannot tell	Yes	No
Han et al. (2019) [27]	Yes	Yes	Yes	Yes	Yes	Yes	Yes
Hartman et al. (2009) [28]	Yes	Yes	Cannot tell	Yes	Yes	No	Yes
Hogan et al. (2018) [29]	Yes	Yes	Yes	Yes	Yes	No	Yes
Johnson et al. (2014) [30]	Yes	Yes	Cannot tell	Cannot tell	Yes	Yes	No
Ong et al. (2018) [31]	Yes	Yes	Yes	Yes	Yes	Cannot tell	No
Rothmann et al. (2016) [32]	Yes	Yes	Yes	Yes	Yes	Yes	No

	Screening questions		3. Non-randomised studies				
First author	S1. Are there clear research questions?	S2. Do the collected data allow to address the research questions?	3.1. Are the participants representative of the target population?	3.2. Are measurements appropriate regarding both the outcome and intervention (or exposure)?	3.3. Are there complete outcome data?	3.4. Are the confounders accounted for in the design and analysis?	3.5. During the study period, is the intervention administered (or exposure occurred) as intended?
Carter et al. (2010) [55]	Yes	Yes	Yes	Yes	Yes	Cannot tell	Yes
Cavarocchi et al. (2019) [38]	Yes	Yes	Yes	Yes	Yes	Yes	Yes
Cedja et al. (2012) [56]	Yes	Yes	Yes	Yes	No	Cannot tell	Yes
deAmorin e Silva (2006) [57]	Yes	Yes	Yes	Yes	Yes	Cannot tell	Yes
Johnson et al. (2009) [46]	Yes	Yes	Yes	Yes	Yes	No	Yes
Karakas et al. (2015) [40]	Yes	Yes	Cannot tell	Yes	Yes	No	Yes
Mastro et al. (2019) [58]	Yes	Yes	Yes	Yes	Yes	Cannot tell	Yes
McGlashan et al. (2017) [4]	Yes	No	Yes	Cannot tell	No	No	Yes
Morel et al. (2020) [47]	Yes	Cannot tell	Cannot tell	Cannot tell	Yes	No	Yes
Thung et al. (2018) [51]	Yes	Yes	Yes	Yes	Yes	Yes	Yes
Tornqvist et al. (2015) [42]	Yes	Yes	Yes	Yes	Yes	No	Yes
Train et al. (2006) [43]	Yes	Yes	Yes	No	Yes	No	Yes
Waitayawinyu & Wankan (2016) [52]	Yes	Yes	Yes	Yes	Yes	Cannot tell	Yes
Williams & Green (2015) [44]	Yes	Yes	Yes	Yes	Yes	Cannot tell	Yes

	Screening questions		4. Quantitative descriptive studies				
First author	S1. Are there clear research questions?	S2. Do the collected data allow to address the research questions?	4.1. Is the sampling strategy relevant to address the research question?	4.2. Is the sample representative of the target population?	4.3. Are the measurements appropriate?	4.4. Is the risk of nonresponse bias low?	4.5. Is the statistical analysis appropriate to answer the research question?
Ashmore et al. (2019) [34]	No	Cannot tell	Yes	Yes	Yes	Cannot tell	Can't tell
Barnea-Goraly et al. (2014) [35]	Yes	Yes	Yes	Yes	Yes	Yes	Yes
Capurso et al. (2020) [36]	Yes	Yes	Yes	Yes	Yes	Yes	Yes
de Bie et al. (2010) [37]	Yes	Yes	Yes	Yes	Yes	Yes	Yes
Durand et al. (2015) [45]	Yes	Yes	Yes	Yes	Yes	Yes	Yes
Fraser et al. (2019) [39]	Cannot tell	Cannot tell	Cannot tell	Yes	Cannot tell	Cannot tell	Cannot tell
Hallowell et al. (2008) [54]	Yes	Yes	Yes	Yes	Yes	Yes	Yes
Nordahl et al. (2016) [48]	Cannot tell	Yes	Yes	Yes	Yes	Yes	Yes
Pressdee et al. (1997) [59]	No	Cannot tell	Yes	Yes	Cannot tell	Yes	Cannot tell
Pua et al. (2020) [49]	Yes	Yes	Yes	Yes	Yes	Yes	Yes
Yamada	Yes	Yes	Yes	Yes	Cannot tell	Yes	Yes

	Screening questions		5. Mixed methods studies				
First author	S1. Are there clear research questions?	S2. Do the collected data allow to address the research questions?	5.1. Is there an adequate rationale for using a mixed methods design to address the research question?	5.2. Are the different components of the study effectively integrated to answer the research question?	5.3. Are the outputs of the integration of qualitative and quantitative components adequately interpreted?	5.4. Are divergences and inconsistencies between quantitative and qualitative results adequately addressed?	5.5. Do the different components of the study adhere to the quality criteria of each tradition of the methods involved?
Szerzak et al. (2016) [50]	Yes	Yes	Yes	Yes	Yes	Cannot tell	Cannot tell

#### Synthesis

Due to clinical and methodological heterogeneity across the included studies, it was not considered feasible to conduct a meta-analysis. Therefore, a narrative synthesis of the key findings was undertaken, this synthesis adopted a textual approach to ‘tell the story’ of the evidence from the included studies [23].

## Results

### Search results

**Fig. 1:**
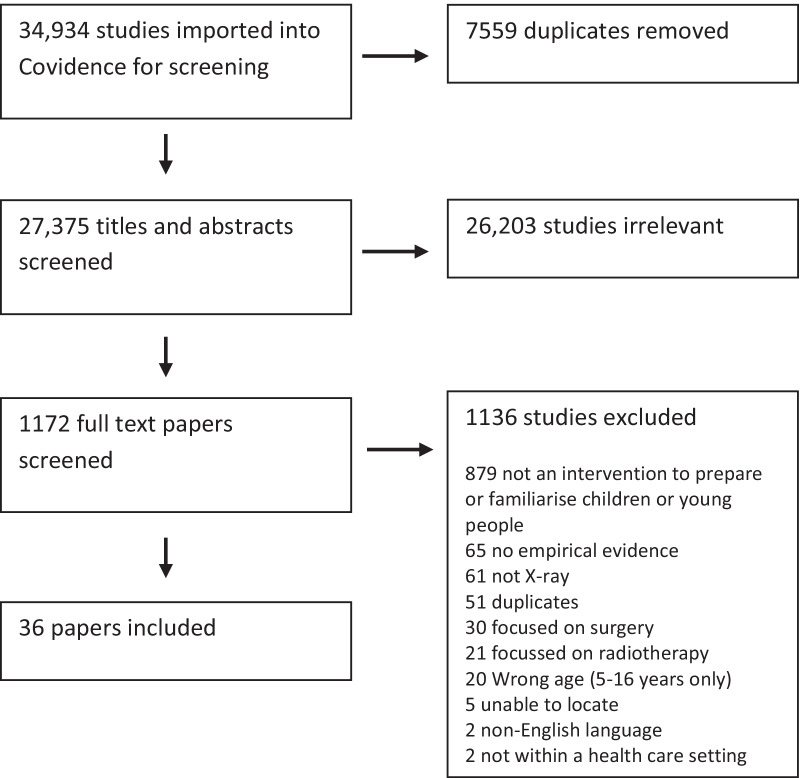
Preferred Reporting Items for Systematic Reviews and Meta-Analyses (PRISMA)

A total of 34,934 articles were identified after the database search. Among those articles, 7559 duplicates were removed. The remaining 27,375 papers were screened independently by two reviewers (LBr, HS) according to their title and abstract. This resulted in 26,203 papers being removed and 1172 papers remaining within the review for full-text screening. Each full-text paper was reviewed independently by two reviewers (LBr, HS) within the review software. This resulted in 1135 papers being excluded (reasons for exclusion included not an intervention to prepare or familiarise children or young people, not empirical evidence, not a radiological procedure, duplicate, radiotherapy, age of children outside the review criteria, non-English language, not within a healthcare setting) and 36 papers being retained for data extraction and quality appraisal. The PRISMA procedure is detailed in Fig. 1.

### Key characteristics of the included studies

There was huge variability in the radiological procedures included in the studies, the foci and delivery of the interventions and methods to prepare, educate or familiarise children and young people, the study designs and the outcomes assessed.

#### The research designs included in the studies

All of the 36 studies used a range of quantitative methods including; 10 randomised controlled trials [24–33], 11 cohort studies [34–44], 3 before and after studies [45, 47], 6 descriptive quantitative studies [48–53], 1 prospective audit [54] and 5 retrospective audits [55–59]. Four of the studies also had a nested qualitative element, to gather views and experiences either through short, structured interviews or open text responses on a questionnaire from children and young people [29, 47, 50] and parents [47].

#### The radiological procedures included in the studies

The majority of the studies focussed exclusively on MRI scans (n = 29) [24, 28, 29, 31, 32, 34–36, 38–42, 45, 47–56, 58, 59]. The other studies focussed on interventions linked to children undergoing intravenous pyelograms (n = 2) [25, 26] voiding cystourethrograms (VCUG) (n = 2) [26], dimercaptosuccinic acid (DMSA) scans (n = 1), chest radiography (n = 1) [27] or interventions linked to multiple radiological procedures, CT, MRI, nuclear medicine and fluoroscopy (n = 3) [30, 44, 46].

#### The interventions to prepare, educate or familiarise children included in the studies

The non-invasive interventions in the studies focussed on different methods of delivery of preparation, education or familiarisation. Some papers included detailed descriptions of how and when a specific intervention was delivered and some only included minimal information of the intervention delivery. The non-invasive interventions included access to technology, facilitated play, the provision of information and opportunities to practice a radiological procedure. The interventions using technology included smartphone applications (n = 4) [30, 34, 44, 49], interactive videos (n = 2) [31, 33], animations (n = 3) [33, 41, 50] and one study focussed on virtual reality information [27]. The most frequent non-invasive intervention described was the opportunity to practice undergoing a procedure, to model what would happen and experience the sensory elements involved in undergoing an MRI scan; these included mock scanners (n = 9) (both toy-sized or pretend full-sized scanners) [24, 35, 37, 47, 48, 51, 53, 55, 57], one study using simulated practice [32] and studies with a focus on play-based learning and preparation (n = 7) [38, 39, 45, 54, 56, 58, 59].

The non-invasive interventions which focussed on the provision of information or education included the use of; educational videos (n = 5) [29, 31, 32, 49, 52], a radiology colouring book (n = 2) [46, 59], a photo-diary/booklet (n = 2) [28, 43], a story-book (n = 2) [26, 54], individualised information provision (n = 2) [25, 57] or a visit to the department to meet staff and watch a child having an MRI scan [40]. Some studies evaluated interventions with multiple elements [32, 35, 36, 42, 58].

The delivery of the non-invasive interventions varied and included play specialists/child life specialists (n = 11) [34, 37–39, 44, 45, 51, 54, 56, 58, 59], parents (n = 6) [26, 28, 35, 46, 48, 49] radiology department staff (n = 2) [32, 35, 47]^.^. Delivery in the other studies was by a paediatrician and medical social worker [24], medical staff [24, 37], volunteers within the department [36], paediatrician and child life specialist [37, 57], paediatric occupational therapist [55], research team member [30, 40, 43], behaviour analyst [48], staff trained in child neurology and behavioural paediatrics [53], nurse [25] or in seven studies, the non-invasive interventions were used by the children in a self-directed manner [27, 29, 41, 44, 46, 50, 52]. In two of the papers, it was not clear who had delivered the intervention [31, 42].

#### The outcomes measured in the studies

The outcomes measured and assessed within the included studies were varied; the outcomes measured within each study are given in Table 4. The most common outcomes were focussed on the completion of a good quality radiological image and these included; image quality (n = 11) [24, 36–38, 42, 52, 56, 58], and successful completion of the procedure (n = 7) [31, 33, 36, 39, 40, 48, 49]. The child orientated outcomes included; child anxiety (n = 8) [27, 28, 32, 44, 46, 47, 50, 51], child distress (n = 4) [25–27, 43], other studies included, child cooperation [25], child information seeking behaviours [25], a child’s need for parental presence [27], child stress [28, 30], child knowledge [29, 41, 50], child relaxation [29], child displaying challenging behaviour [30], child’s confidence in staying still [33] and child compliance [44]. The measurement and definition of what constituted ‘compliance’ or the ‘successful completion’ of a procedure was often not included within the papers. Some outcomes focussed on children’s engagement with the interventions these included a child’s ability to undergo the training session [37], helpfulness of information [34, 41], ease of use of the intervention [34] and enjoyability of the resource [34].

**Table 4:** The different outcomes measured within the evidence

	Outcomes linked to children accessing procedural information	Outcomes linked to children gaining procedural understanding and knowledge			Outcomes linked to children’s application of information and knowledge on their procedural experiences and outcomes							
Paper	Useability/perceptions of using the intervention	Child’s reported knowledge and understanding	Parents’ reported knowledge and understanding	Children’s ability to rehearse/act out key elements of the procedure	Parental anxiety	Child anxiety or distress	Parent or child satisfaction	Scan quality	Scan completion	Scan length	Need for sedation	Need for GA
XAshmore et al. (2019) [34]	X	X			X							X
Barnea-Goraly et al. (2014) [35]								X	X			
Bharti et al. (2016) [24]											X	X
Capurso et al. (2020) [36]								X	X			
de Bie et al. (2010) [37]								X	X			
Carter et al. (2010) [55]												X
Cavarocchi et al. (2019) [38]								X			X	
Cedja et al. (2012) [56]									X		X	
de Amorim e Silva et al. (2006) [57]									X			X
Durand et al. (2015) [45]									X			X
Fegley (1988) [25]			X									
Fraser (2019) [39]									X		X	
Gebarski et al. (2013) [26]						X						
Hallowell et al. (2008) [54]								X	X			
Han et al. (2019) [27]						X	X					
Hartmann et al. (2009) [28]						X	X					
Hogan et al. (2018) [29]			X									
Johnson et al. (2009) [46]	X		X			X						
Johnson et al. (2014) [30]						X				X		
Karakas et al. (2015) [40]						X						
Mastro et al. (2019) [58]								X		X		
McGlashan et al. (2017) [4]	X		X			X						
Morel (2020) [47]						X						
Nordahl (2016) [48]									X		X	
Ong et al. (2018) [31]												X
Pressdee et al. (1997) [59]												X
Pua et al. (2020) [49]									X			
Rothman et al. (2016) [32]						X						X
Szeszak et al. (2016) [50]	X		X			X						
Thung (2018) [51]												X
Tornqvist et al. (2015) [42]						X	X				X	X
Train et al. (2006) [43]						X	X				X	
Utama et al. (2019)									X			X
Waitayawinyu & Wankan (2016) [52]									X		X	
Williams & Greene (2015) [44]						X				X		
Yamada et al. (2020) [53]									X			

The parent-focussed outcomes included parental satisfaction [27], process difficulty score [27], parental anxiety [28, 43] and parental satisfaction [42].

The outcomes which were focussed on procedural time, costs and the need for additional procedural support also varied across the studies; eight studies included the need for sedation [24, 32, 38, 42, 43, 45, 51, 56], nine studies measured the need for a general anaesthetic [31, 33, 42, 45, 52, 55–57, 59], other outcomes measured included additional time taken to be ready for imaging [44], procedure time [27, 30, 49, 58], cost savings [39, 42, 58] and additional attempts to complete a successful scan [27, 35].

#### Reported impact and value of the interventions and methods to prepare, educate or familiarise children for radiological procedures

The evidence shows that the introduction of additional preparation, education or familiarisation interventions have a positive reported impact on children’s anxiety and distress levels and increase the number of radiological procedures, particularly MRI, which are completed without sedation or anaesthesia. However, due to the variability in outcomes, measures and research designs we are unable to report and conclude on the overall effectiveness of interventions. The reported impact and value of the interventions will be discussed according to the following outcomes: children’s use and perceptions of the interventions, children’s and parents’ knowledge and understanding of the radiological procedure, completion of the radiological procedure, quality of the scan/image obtained, children’s anxiety and distress levels and children’s and parents’ satisfaction (see Table 4).

### Children’s use and perceptions of the interventions and methods to prepare, educate or familiarise them before their radiological procedure

Several of the studies examined children’s and parents’ views of their child using the intervention [34, 41, 46, 50]. In one study, 96% (n = 22) of children reported that they liked the MRI animation they saw and 100% (n = 23) liked the way the animation looked and sounded [50]. While most feedback about watching the MRI animation before the procedure was positive, some children in McGlashan et al.’s (2017) study wanted more realistic and louder noises within the animated video. Parent proxy reports showed that their children found using a preparation smartphone application enjoyable (median 8.5), useful (median 8) and easy to use (median 10) [34] and 92% (n = 155) of parents reported that their child was ‘pleased’ to have had access to a colouring book to help prepare them [46]. One study asked health professionals for their views about children using a smartphone application to prepare them for an MRI and all reported that the intervention was useful for children to access and use prior to their procedure [34].

### Impact and value of the preparation, education or familiarisation interventions on children’s and parents’ knowledge and understanding of the radiological procedure

Children undergoing an MRI have been shown to have an improved understanding of their procedure after watching an instructional video compared to controls [29] and after watching an educational animation [41, 50].

Parents have also reported an improved understanding of their child’s radiological procedure after their child used a colouring book to help prepare them [46] and after their child interacted with a smartphone application and booklet before their MRI scan [34].

### Impact and value of the preparation, education or familiarisation interventions on radiological scan quality

All the studies (n = 6) which measured the impact of an intervention on the quality of the scan/image obtained showed a positive impact, with the majority of these focussing on the use of mock scanners, 92% (n = 204) of children had usable MRI scans after accessing a mock scanner [35], 90% (n = 81) of MRI scans were of diagnostic quality after children accessed a mock scanner [37], 100% (n = 891) of brain MRI images were of a sufficient quality after children accessed a toy ‘kitten’ scanner [38], 96% (n = 218) of scans were of a diagnostic quality on children who practised their scan [54], 100% (n = 17) of scans (T1-weighted images) met quality assurance for acceptable motion artefact and 94% (n = 16) of children achieved a high-quality diffusion-weighted image after using a mock scanner [48]. After play-based sessions, 97% (n = 121) of children who accessed a medical play session including a mock scanner and information achieved a good quality MRI image [58] and 92% (n = 61) of children achieved clinical diagnostic MRI scans after play-based simulation [36]^.^

### Impact and value of the preparation, education or familiarisation interventions on radiological scan completion

The studies report a mainly positive impact of the intervention on radiological scan completion along with a reduced need for additional procedural support. The reported impacts include: increased first-time scan completion (n = 3) [27, 31, 35], successful scan completion (n = 2) [49, 53], reduced time of scan completion (n = 2) [44, 58], reduced preparation time (n = 1) [44], reduced use of sedation (n = 9) [24, 32, 38, 42, 43, 45, 51, 52, 56], reduced need for a general anaesthetic (n = 9) [32, 34, 42, 45, 54–57, 59] and improved compliance during scan procedures (n = 2) [40, 44]. Some studies showed no effect of an intervention on scan completion, particularly in regard to the need for a general anaesthetic [31, 33].

There was limited information within the papers to accompany what exactly constituted ‘compliance’ [44] and ‘successful completion’ [36, 39, 48]. Many of the studies which note a statistically significant reduction of the use of sedation and anaesthesia have small sample sizes [24].

### Impact and value of the preparation, education or familiarisation interventions on children’s and parents’ anxiety and distress

The evidence indicates that interventions and methods used before a radiological procedure can help reduce children’s anxiety before and also during a radiological procedure. However, there are difficulties in drawing together the evidence as the studies use different terms and approaches to measuring anxiety and distress with many using locally developed unvalidated scales and many studies only involving small sample sizes or no comparison/ control group.

The majority of the studies focussed on children undergoing MRI scans and showed that watching an educational animated video helped children feel less ‘nervous’ before their MRI scan [41, 50] and ‘more confident’ and ‘less frightened’ during their scan [50]. Children exposed to a teddy-bear-sized mock MRI scanner had lower anxiety levels before their MRI examination [47] and training with a mock scanner alongside coping strategies such as deep breathing or guided imagery was shown to reduce children’s procedural anxiety [51]. Interestingly, this study found that those children who had higher baseline levels of procedural anxiety did not benefit from the training [51]. Other studies have shown decreased distress and higher ‘tolerance’ prior to undergoing an VCUG for children who viewed a storybook [26] and decreased distress as rated on the Observation Scale of Behavioural Distress (OSBD) for children undergoing a chest radiograph who had used VR [27]. A further study showed that a photo booklet depicting a child having a DMSA scan and an information guide for parents decreased children’s distress levels before their scan [43]. A smartphone application developed to educate a cohort of children prior to having a range of medical imaging procedures was shown to reduce children’s anxiety levels [44]. Two studies reported null findings, showing that children’s procedural 93anxiety was not reduced after using a photo book to familiarise and prepare them prior to an MRI scan [28] or after using a colouring book to prepare them prior to a CT, fluoroscopy, ultrasound or nuclear medicine procedure [30]. While no statistical significance was seen between the control and intervention group, parents (57%, n = 95) reported that they felt the colouring book had made their child ‘less worried’ about the procedure [30]. One study demonstrated that a smartphone application helped to reduce children with Autism Spectrum Conditions (ASC) anxiety by measuring physiological parameters (blood pressure, pulse) and assessing rates of ‘challenging behaviours’ to judge that a smartphone application helped prior to undergoing MRI, CT scan, plain radiograph and nuclear medicine [30].

Some studies evaluating interventions linked to MRI scans focussed on parental anxiety as an outcome, showing a reduction in parental anxiety after their child had accessed a smartphone application pre-scan [34] or a significant reduction in parental anxiety after access to a multi-element intervention (instructional booklet, video and simulation practice) prior to an MRI scan [32].

### Impact of the preparation, education or familiarisation interventions on children’s and parents’ satisfaction of undergoing radiological procedures

The studies (n = 4) which measured the impact of an intervention on parents’ satisfaction related to a radiological procedure, show mixed results. Studies showed significantly higher parent-reported procedural satisfaction in a cohort of children who accessed a photo booklet before a DMSA scan [43], in parents whose child accessed virtual reality prior to a chest radiograph [27] and a nonsignificant trend for greater satisfaction in parents whose child accessed a photo diary before an MRI scan [28]. One study showed no significant difference in parents’ reported satisfaction after their child accessed a multi-element preparation program before an MRI compared to controls [42].

## Discussion

The evidence suggests that interventions to prepare, educate or familiarise children and young people prior to their radiological procedures have value in improving children’s knowledge, increasing the opportunity to gain good quality scans, reducing children’s anxiety and reducing the need for sedation and general anaesthetic. What is less clear is which elements and modes of delivery of an intervention are most valuable for improving the outcomes of children attending for radiological procedures. Many of the interventions included complex and interrelated components and there was huge disparity between studies relating to the resource and staff input required to deliver an intervention. The complexity and heterogeneity of the interventions and evaluation is exacerbated by the range of outcomes measured and reported. This results in challenges in drawing together a clear understanding of the value and impact of interventions to improve children’s experiences of undergoing a radiological procedure. This led us to consider the challenges and opportunities linked to amassing an evidence base to underpin the development of interventions to prepare, educate and familiarise children prior to radiological procedures.

**Fig. 2:**
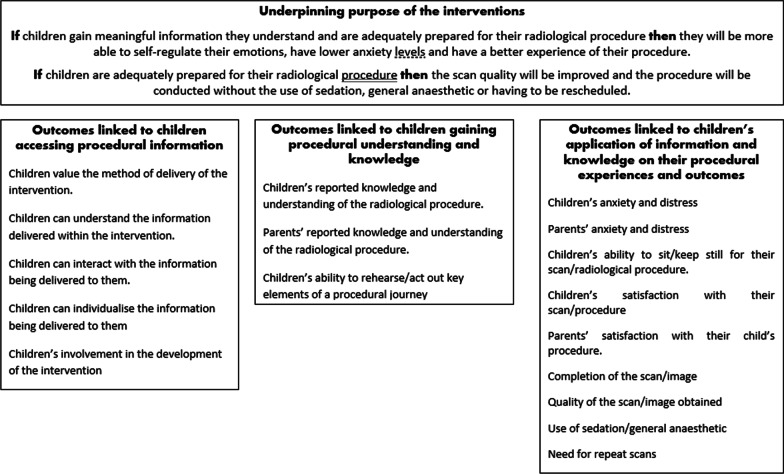
Mapping the outcomes of interventions alongside a health literacy framework

In examining the findings from this review, we conclude that the use of a health literacy framework is useful to consider the focus, delivery and potential outcomes of such interventions. The need for child-centred interventions and approaches to improve children’s health literacy is well recognised [8, 60], with literature increasingly showing that while improving children’s ability to access, understand and evaluate health information and services is important, health literacy also has an important role in empowering children to become more engaged in shaping and making decisions and choices about their healthcare [61, 62]. We will consider the review findings within three elements of health literacy, accessing procedural information, gaining procedural understanding and knowledge and lastly the application of knowledge and understanding to shape a child’s behaviour and experiences during their radiological procedure (Fig. 2).

This review highlights how children valued the focussed delivery of engaging interventions, enabling them to access useful information and gain knowledge. It is not clear in the papers we reviewed how involved children had been in the development of the interventions and not all studies asked children their opinions of using and accessing information within the various interventions.

The review highlights that the interventions improved children’s and parents’ reported knowledge and understanding of their radiological procedure. However, knowledge and understanding were only included as outcomes in a few studies.

The main focus of interventions and methods was on reducing children’s anxiety and improving their ability to sit or lie still to facilitate a good quality scan without the use of sedation or general anaesthetic. However, there are a lack of first-hand accounts from children within the evidence to help determine which specific elements of the interventions are most valuable to children and how the content and delivery translates to children being able to shape their procedural experiences by self-regulating their emotions and enacting their gained knowledge or practice into sitting still for their procedure. There is a need for evaluations to place greater emphasis on children’s self-reports and procedural experiences as an important outcome alongside scan quality and length of radiological procedure as metrics. There is currently a lack of child voice to shape the important outcomes and metrics of interventions to help inform, educate and prepare children prior to radiological procedures. The need to include children as equal voices in the development of core outcomes for interventional studies is gaining increased awareness to ensure measured outcomes are clinically meaningful [63].

The lack of consistency across the focus, delivery and outcomes of non-invasive interventions to prepare, educate and familiarise children before a radiological procedure has resulted in challenges for the speciality in drawing together a clear understanding of which interventions offer the best option for use within radiology departments. This paper has attempted to outline a framework of the core outcomes to be considered in the future development, evaluation and reporting of non-invasive interventions to prepare, educate and familiarise children before a radiological procedure. The authors conclude that integral to any further development, implementation and evaluation, radiology professionals and researchers carefully consider this framework to amass a core of evidence which would enable comparison between different interventions and inform evidence-based decision-making.

## Limitations of the scoping review

There are several limitations to this work which should be considered when interpreting the findings. The scoping review findings are informed by English-language papers only and therefore evidence in papers written in other languages was excluded. The findings of the review are limited to non-invasive interventions to prepare, educate and familiarise children aged 5 years and above.

## Conclusion

Interventions and methods to prepare, educate or familiarise children and young people prior to their radiological procedures have value in improving children’s knowledge and reducing their anxiety while increasing the opportunity to gain good quality scans without the need for sedation and general anaesthetic. However, there is insufficient consistency within the evidence to recommend implementation. Many of the interventions include complex and interrelated components, there was huge disparity between the resource and staff input involved in delivering an intervention and wide variability in the outcomes used to judge impact and value. There is a need for consistency of measures and outcomes across evaluation studies and for children to help shape the development of core outcomes for interventional studies.

## Supplementary Information

**Additional file 1: Appendix A** - Overview of search terms used framed by Population, Concept and Context. **Appendix B** - Detailed search strategy. **Appendix C** - Data extraction and detailed charting table.

## Data Availability

All data generated or analysed during this study are included in this published article.

## Abbreviations

CT: Computed tomography
MMAT: The Mixed Methods Appraisal Tool
MRI: Magnetic resonance imaging
PRISMA: Preferred Reporting Items for Systematic Reviews and Meta-Analyses
US: Ultrasound
