# Supplementary material for: Interventions and methods to prepare, educate or familiarise children and young people for radiological procedures: a scoping review
Source: Insights Imaging. 2022 Sep 5;13:146. doi: 10.1186/s13244-022-01278-5 (PMC9445139; doi:10.1186/s13244-022-01278-5)
Supplement: Supplementary file 1 — Additional file 1: Appendix A - Overview of search terms used framed by Population, Concept and Context. Appendix B - Detailed search strategy. Appendix C - Data extraction and detailed charting table. [file 13244_2022_1278_MOESM1_ESM.pdf]

ELECTRONIC SUPPLEMENTARY MATERIAL

Interventions and methods to prepare, educate or familiarise children and young people for radiological procedures: a scoping review

Appendix A: Overview of search terms used framed by Population, Concept and Context

**Population**	**adolescen*,child*, "young person", "young people", teenager*, youth*, pediatric*, paediatric***
**Context**	**procedur*, invasive procedure*, noninvasive procedure*, X-Ray*, Magnetic Resonance Imaging, MRI, Tomography, U/S scan, Neuroimaging, radionuclide Imaging, Emission-Compute, magnetic resonance imag*, radiolog*, radiograph*, ultrasound* barium, DMSA, dimercapto succinic acid, CT scan* or CAT scan*, Computed tomography, urodynamics, voiding cystourethrogram, imaging, nuclear medicine, PET scan*, positron emission tomography, Fluoroscopy, DEXA scan*, dual energy X-ray absorptiometry, EOS scan*, EOS imaging, LINAC, linear accelerator, IVP, intravenous pyelogram***

**Concept**	**child life therap*, plaything*, play therap*, play specialist*, role play*, clown doctor*, art therap*, play, animation*, comic*, virtual reality, VR, augmented reality, AI, mobile applications, DVD, leaflet*, cartoon*, book*, storybook*, game*, website*, storyboard*, app*, ipad*, toy scanner* toy MRI, mock scanner*, mock MRI, lego, photobook*, mock or video*, therapeutic, mHealth, online system*, picture*, internet, online, patient information, film*, educational intervention, psychological, mobile applications, telemedicine, video games, games, patient education, pamphlets, prepar*, educat*, information,**

Appendix B: Full Search Terminologies Used

MEDLINE (Ovid) 1946-Feb 2021

1. "procedure*".m_titl.
2. (adolescen* or child* or "young person" or "young people" or teenager* or youth* or pediatric* or paediatric*).m_titl.
3. 1 and 2
4. exp Radiography/
5. exp X-Rays/
6. exp Magnetic Resonance Imaging/
7. exp Radiotherapy/
8. exp Tomography, X-Ray Computed/
9. exp Radiology/
10. exp Urodynamics/
11. exp Neuroimaging/
12. exp Radionuclide Imaging/
13. exp Tomography, Emission-Computed/
14. 4 or 5 or 6 or 7 or 8 or 9 or 10 or 11 or 12 or 13
15. exp Play Therapy/
16. exp Art Therapy/
17. exp Role Playing/
18. exp AUDIOVISUAL AIDS/
19. exp Mobile Applications/
20. exp Telemedicine/
21. exp VIDEO GAMES/ or exp GAMES, RECREATIONAL/
22. exp Patient Education as Topic/
23. exp PATIENT EDUCATION HANDOUT/
24. exp INTERNET/
25. exp Augmented Reality/
26. exp Pamphlets/
27. 15 or 16 or 17 or 18 or 19 or 20 or 21 or 22 or 23 or 24 or 25 or 26
28. 14 and 27
29. ((radiotherap* or x-ray* or MRI or MR or "magnetic resonance imaging" or radiolog* or radiograph* or ultrasound* or barium or DMSA or dimercapto succinic acid or CT scan* or CAT scan* or Computed tomography or urodynamics or voiding cystourethrogram or imaging or nuclear medicine or PET scan* or positron emission tomography or Fluoroscopy or DEXA scan* or dual energy X-ray absorptiometry or EOS

scan* or EOS imaging or LINAC or linear accelerator or IVP or intravenous pyelogram*) adj7 (child life therap* or plaything* or play therap* or play specialist* or role play* or clown doctor* or art therap* or play or animation* or comic or comics or virtual reality or VR or augmented reality or AI or mobile applications or DVD or leaflet* or cartoon* or book* or storybook* or game* or website* or storyboard* or app or apps or ipad* or toy scanner* or toy MRI or mock scanner* or mock MRI or lego or photobook* or mock or video* or therapeutic or mHealth or online system* or picture* or internet or patient information or film* or educational intervention or psychological)).ti,ab,kw.
30. 28 or 29
31. (educat* or prepara* or prepare* or inform* or familiari* or procedur* or impact or evaluat* or effective* or efficacy).ti,ab.
32. 30 and 31
33. limit 32 to (english language and "all child (0 to 18 years)")
34. (adolescen* or child* or "young person" or "young people" or teenager* or youth* or pediatric* or paediatric*).ti,ab,kw.
35. 32 and 34
36. limit 35 to english language
37. 33 or 36
38. 3 and 14
39. 37 or 38
40. limit 39 to (case reports or clinical conference or comment or consensus development conference or consensus development conference, nih or editorial or letter or news or newspaper article)
41. 39 not 40
42. limit 41 to english language

Cochrane (CENTRAL) Issue searched Feb 2021

#1 procedure*:ti
#2 (adolescen* or child* or "young person" or "young people" or teenager* or youth* or pediatric* or paediatric*):ti
#3 #1 AND #2
#4 MeSH descriptor: [Radiography] explode all trees
#5 MeSH descriptor: [X-Rays] explode all trees
#6 MeSH descriptor: [Magnetic Resonance Imaging] explode all trees
#7 MeSH descriptor: [Radiotherapy] explode all trees
#8 MeSH descriptor: [Tomography, X-Ray Computed] explode all trees
#9 MeSH descriptor: [Radiology] explode all trees
#10 MeSH descriptor: [Urodynamics] explode all trees
#11 MeSH descriptor: [Neuroimaging] explode all trees

#12 MeSH descriptor: [Radionuclide Imaging] explode all trees
#13 MeSH descriptor: [Tomography, Emission-Computed] explode all trees
#14 {OR #4-#13}
#15 MeSH descriptor: [Play Therapy] explode all trees
#16 MeSH descriptor: [Art Therapy] explode all trees
#17 MeSH descriptor: [Role Playing] explode all trees
#18 MeSH descriptor: [Audiovisual Aids] explode all trees
#19 MeSH descriptor: [Mobile Applications] explode all trees
#20 MeSH descriptor: [Telemedicine] explode all trees
#21 MeSH descriptor: [Video Games] explode all trees
#22 MeSH descriptor: [Games, Recreational] explode all trees
#23 MeSH descriptor: [Patient Education as Topic] explode all trees
#24 MeSH descriptor: [Patient Education Handout] explode all trees
#25 MeSH descriptor: [Internet] explode all trees
#26 MeSH descriptor: [Augmented Reality] explode all trees
#27 MeSH descriptor: [Pamphlets] explode all trees
#28 {OR #15-#27}
#29 #14 AND #28
#30 ((radiotherap* or x-ray* or MRI or MR or "magnetic resonance imaging" or radiolog* or radiograph* or ultrasound* or barium or DMSA or "dimercapto succinic acid" or "CT scan" or "CT scanner" or "CAT scan" or "CAT scanner" or "Computed tomography" or urodynamics or "voiding cystourethrogram" or imaging or nuclear medicine or "PET scan" or "PET scanner" or "positron emission tomography" or Fluoroscopy or "DEXA scan" or "DEXA scanner" or "dual energy X-ray absorptiometry" or "EOS scan" or "EOS scanner" or "EOS imaging" or LINAC or "linear accelerator" or IVP or "intravenous pyelogram") NEAR/7 ("child life therapy" or "child life therapies" or plaything* or "play therapy" or "play therapies" or "play specialist" or "play specialists" or "role play" or "clown doctor" or "clown doctors" or "art therapy" or "art therapies" or play or animation* or comic or comics or "virtual reality" or VR or "augmented reality" or AI or "mobile applications" or DVD or leaflet* or cartoon* or book* or storybook* or game* or website* or storyboard* or app or apps or ipad* or "toy scanner" or "toy scan" or toy MRI or "mock scanner" or "mock scan" or "mock MRI" or lego or photobook* or mock or video* or therapeutic or mHealth or "online system*" or picture* or internet or "patient information" or film* or "educational intervention" or psychological)):ti,ab
#31 #29 OR #30
#32 (educat* or prepara* or prepare* or inform* or familiari* or procedur* or impact or evaluat* or effective* or efficacy):ti,ab
#33 #31 AND #32
#34 (adolescen* or child* or "young person" or "young people" or teenager* or youth* or pediatric* or paediatric*):ti,ab
#35 #33 AND #34
#36 #3 AND #14
#37 #35 OR #36

CINAHL EBSCOHOST 1950 – Feb 2021

S1 TI procedure*
S2 (MH "Invasive Procedures+")
S3 (MH "Noninvasive Procedures")
S4 S1 OR S2 OR S3
S5 TI (adolescen* OR child* OR "young person" OR "young people" OR teenager* OR youth* OR pediatric* OR paediatric*)
S6 S4 AND S5
S7 (MH "Radiography+")
S8 (MH "X-Rays")
S9 (MH "Magnetic Resonance Imaging+")
S10 (MH "Radiotherapy+")
S11 (MH "Tomography, Spiral Computed+")
S12 (MH "Urodynamics")
S13 (MH "Neuroradiography+")
S14 (MH "Radionuclide Imaging+")
S15 (MH "Tomography, Emission-Computed+")
S16 S7 OR S8 OR S9 OR S10 OR S11 OR S12 OR S13 OR S14 OR S15
S17 (MH "Play Therapy")
S18 (MH "Art Therapy")
S19 (MH "Role Playing")
S20 (MH "Audiovisuals")
S21 (MH "Mobile Applications")
S22 (MH "Telemedicine")
S23 (MH "Video Games+")
S24 (MH "Games+")
S25 (MH "Patient Education")
S26 (MH "Preoperative Education")
S27 (MH "Internet+")
S28 (MH "Augmented Reality")
S29 (MH "Pamphlets")
S30 MH "Computer Simulation+"
S31 (MH "Parenting Education")
S32 S17 OR S18 OR S19 OR S20 OR S21 OR S22 OR S23 OR S24 OR S25 OR S26 OR S27 OR S28 OR S29 OR S30 OR S31

S33 S16 AND S32

S34 TI (((radiotherap* OR x-ray* OR MRI OR MR OR "magnetic resonance imaging" OR radiolog* OR radiograph* OR ultrasound* OR barium OR DMSA OR "dimercapto succinic acid" OR "CT scan*" OR "CAT scan*" OR "Computed tomography" OR urodynamics OR "voiding cystourethrogram" OR "imaging or nuclear medicine" OR "PET scan*" OR "positron emission tomography" OR Fluoroscopy OR "DEXA scan*" OR "dual energy X-ray absorptiometry" OR "EOS scan*" OR "EOS imaging" OR LINAC OR "linear accelerator" OR IVP OR "intravenous pyelogram*") N7 ("child life therap*" OR plaything* OR "play therap*" OR "play specialist*" OR "role play*" OR "clown doctor*" OR "art therap*" OR play OR animation* OR comic OR comics OR "virtual reality" OR VR OR "augmented reality" OR AI OR "mobile applications" OR DVD OR leaflet* OR cartoon* OR book* OR storybook* OR game* OR website* OR storyboard* OR app OR apps OR ipad* OR "toy scanner*" OR "toy MRI" OR "mock scanner*" OR "mock MRI" OR lego OR photobook* OR mock OR video* OR therapeutic OR mHealth OR "online system*" OR picture* OR internet OR "patient information" OR film* OR "educational intervention" OR psychological))) OR AB (((radiotherap* or x-ray* or MRI or MR or "magnetic resonance imaging" OR radiolog* or radiograph* or ultrasound* or barium or DMSA or "dimercapto succinic acid" or "CT scan*" or "CAT scan*" or "Computed tomography" or urodynamics or "voiding cystourethrogram" or "imaging or nuclear medicine" or "PET scan*" or "positron emission tomography" or Fluoroscopy or "DEXA scan*" or "dual energy X-ray absorptiometry" or "EOS scan*" or "EOS imaging" or LINAC or "linear accelerator" or IVP or "intravenous pyelogram*") N7 ("child life therap*" or plaything* or "play therap*" or "play specialist*" or "role play*" or "clown doctor*" or "art therap*" or play or animation* or comic or comics or "virtual reality" or VR or "augmented reality" or AI or "mobile applications" or DVD or leaflet* or cartoon* or book* or storybook* or game* or website* or storyboard* or app or apps or ipad* or "toy scanner*" or "toy MRI" or "mock scanner*" or "mock MRI" or lego or photobook* or mock or video* or therapeutic or mHealth or "online system*" or picture* or internet or "patient information" or film* or "educational intervention" or psychological)))

S35 S33 OR S34

S36 TI ((educat* or prepara* or prepare* or inform* or familiari* or procedur* or impact or evaluat* or effective* or efficacy)) OR AB ((educat* or prepara* or prepare* or inform* or familiari* or procedur* or impact or evaluat* or effective* or efficacy))

S37 S35 AND S36

S38 S35 AND S36 Limiters - Age Groups: All Infant, All Child

S39 TI (adolescen* or child* or "young person" or "young people" or teenager* or youth* or pediatric* or paediatric*)) OR AB (adolescen* or child* or "young person" or "young people" or teenager* or youth* or pediatric* or paediatric*))

S40 S37 AND S39

S41 S38 OR S40

S42 S6 AND S16

S43 S41 OR S42

S44 S41 OR S42

PSYCINFO EBSCOHOST 1950 – Feb 2021

S1 TI procedure*
S2 TI (adolescen* OR child* OR "young person" OR "young people" OR teenager* OR youth* OR pediatric* OR paediatric*)
S3 S1 AND S2
S4 (((((DE "Roentgenography") OR (DE "Magnetic Resonance Imaging" OR DE "Diffusion Tensor Imaging" OR DE "Functional Magnetic Resonance Imaging")) OR (DE "Tomography" OR DE "Magnetic Resonance Imaging" OR DE "Positron Emission Tomography" OR DE "Single Photon Emission Computed Tomography")) OR (DE "Radiology")) OR (DE "Neuroimaging" OR DE "Encephalography")
S5 ((((((((((DE "Play Therapy") OR (DE "Role Playing" OR DE "Pretend Play" OR DE "Doll Play")) OR (DE "Art Therapy" OR DE "Creative Arts Therapy")) OR (DE "Educational Audiovisual Aids")) OR (DE "Mobile Applications")) OR (DE "Telemedicine"))) OR (DE "Games" OR DE "Brain Training" OR DE "Chess" OR DE "Childrens Recreational Games" OR DE "Computer Games" OR DE "Entrapment Games" OR DE "Non Zero Sum Games" OR DE "Prisoners Dilemma Game" OR DE "Role Playing Games" OR DE "Simulation Games")) OR (DE "Client Education")) OR (DE "Internet")) OR (DE "Augmented Reality")
S6 S4 AND S5
S7 TI ((((radiotherap* OR x-ray* OR MRI OR "magnetic resonance imaging" OR radiolog* OR radiograph* OR ultrasound* OR barium OR DMSA OR "dimercapto succinic acid" OR "CT scan*" OR "CAT scan*" OR "Computed tomography" OR urodynamics OR "voiding cystourethrogram" OR "imaging or nuclear medicine" OR "PET scan*" OR "positron emission tomography" OR Fluoroscopy OR "DEXA scan*" OR "dual energy X-ray absorptiometry" OR "EOS scan*" OR "EOS imaging" OR LINAC OR "linear accelerator" OR IVP OR "intravenous pyelogram*") N7 ("child life therap*" OR plaything* OR "play therap*" OR "play specialist*" OR "role play*" OR "clown doctor*" OR "art therap*" OR play OR animation* OR comic OR comics OR "virtual reality" OR "augmented reality" OR "mobile applications" OR DVD OR leaflet* OR cartoon* OR book* OR storybook* OR game* OR website* OR storyboard* OR app OR apps OR ipad* OR "toy scanner*" OR "toy MRI" OR "mock scanner*" OR "mock MRI" OR lego OR photobook* OR mock OR video* OR therapeutic OR mHealth OR "online system*" OR picture* OR internet OR "patient information" OR film* OR "educational intervention" OR psychological)))) OR AB ((((radiotherap* OR x-ray* OR MRI OR "magnetic resonance imaging" OR radiolog* OR radiograph* OR ultrasound* OR barium OR DMSA OR "dimercapto succinic acid" OR "CT scan*" OR "CAT scan*" OR "Computed tomography" OR urodynamics OR "voiding cystourethrogram" OR "imaging or nuclear medicine" OR "PET scan*" OR "positron emission tomography" OR Fluoroscopy OR "DEXA scan*" OR "dual energy X-ray absorptiometry" OR "EOS scan*" OR "EOS imaging" OR LINAC OR "linear accelerator" OR IVP OR "intravenous pyelogram*") N7 ("child life therap*" OR plaything* OR "play therap*" OR "play specialist*" OR "role play*" OR "clown doctor*" OR "art therap*" OR play OR animation* OR comic OR comics OR "virtual reality" OR "augmented reality" OR "mobile applications" OR DVD OR leaflet* OR cartoon* OR book* OR storybook* OR game* OR website* OR storyboard* OR app OR apps OR ipad* OR "toy scanner*" OR "toy MRI" OR "mock scanner*" OR "mock MRI" OR lego OR photobook* OR mock OR video* OR therapeutic OR mHealth OR "online system*" OR picture* OR internet OR "patient information" OR film* OR "educational intervention" OR psychological))))
S8 S6 OR S7 Expanders - Apply equivalent subjects
S9 TI ((educat* or prepara* or prepare* or inform* or familiari* or procedur* or impact or evaluat* or effective* or efficacy) OR AB ((educat* or prepara* or prepare* or inform* or familiari* or procedur* or impact or evaluat* or effective* or efficacy) Expanders - Apply equivalent subjects
S10 S8 AND S9 Expanders - Apply equivalent subjects

S11 S8 AND S9 Expanders - Apply equivalent subjects
S12 S8 AND S9 Limiters - Age Groups: Childhood (birth-12 yrs), Adolescence (13-17 yrs)
S13 TI ((adolescen* or child* or "young person" or "young people" or teenager* or youth* or pediatric* or paediatric*)) OR AB ((adolescen* or child* or "young person" or "young people" or teenager* or youth* or pediatric* or paediatric*))
S14 S11 AND S13
S15 S12 OR S14
S16 S3 AND S4
S17 S15 OR S16

WOS Feb 2021

#1 TI=(radiotherap* OR x-ay* OR MRI OR MR OR "magnetic resonance imaging" OR radiolog* OR radiograph* OR ultrasound* OR barium OR DMSA OR "dimercapto succinic acid" OR "CT scan*" OR "CAT scan*" OR "Computed tomography" OR urodynamics OR "voiding cystourethrogram" OR "imaging or nuclear medicine" OR "PET scan*" OR "positron emission tomography" OR Fluoroscopy OR "DEXA scan*" OR "dual energy X-ray absorptiometry" OR "EOS scan*" OR "EOS imaging" OR LINAC OR "linear accelerator" OR IVP OR "intravenous pyelogram*") AND TI=(procedure*) AND TI=(adolescen* or child* or "young person" or "young people" or teenager* or youth* or pediatric* or paediatric*)

#2 TS= ((radiotherap* OR x-ray* OR MRI OR MR OR "magnetic resonance imaging" OR radiolog* OR radiograph* OR ultrasound* OR barium OR DMSA OR "dimercapto succinic acid" OR "CT scan*" OR "CAT scan*" OR "Computed tomography" OR urodynamics OR "voiding cystourethrogram" OR "imaging or nuclear medicine" OR "PET scan*" OR "positron emission tomography" OR Fluoroscopy OR "DEXA scan*" OR "dual energy X-ray absorptiometry" OR "EOS scan*" OR "EOS imaging" OR LINAC OR "linear accelerator" OR IVP OR "intravenous pyelogram*") NEAR/7 ("child life therap*" OR plaything* OR "play therap*" OR "play specialist*" OR "role play*" OR "clown doctor*" OR "art therap*" OR play OR animation* OR comic OR comics OR "virtual reality" OR VR OR "augmented reality" OR AI OR "mobile applications" OR DVD OR leaflet* OR cartoon* OR book* OR storybook* OR game* OR website* OR storyboard* OR app OR apps OR ipad* OR "toy scanner*" OR "toy MRI" OR "mock scanner*" OR "mock MRI" OR lego OR photobook* OR mock OR video* OR therapeutic OR mHealth OR "online system*" OR picture* OR internet OR "patient information" OR film* OR "educational intervention" OR psychological))

#3TS=(educat* or prepara* or prepare* or inform* or familiari* or procedur* or impact or evaluat* or effective* or efficacy)

#4 TS=(adolescen* or child* or "young person" or "young people" or teenager* or youth* or pediatric* or paediatric*)

#5 #4 AND #3 AND #2

#6 #5 OR #1 Refined by: LANGUAGES: (ENGLISH) AND [excluding] DOCUMENT TYPES: (MEETING ABSTRACT OR EDITORIAL MATERIAL OR LETTER OR PROCEEDINGS PAPER OR BOOK CHAPTER)

(((radiotherap* OR x-ray* OR MRI OR "magnetic resonance imaging" OR radiolog* OR radiograph* OR ultrasound* OR barium OR DMSA OR "dimercapto succinic acid" OR "CT scan*" OR "CAT scan*" OR "Computed tomography" OR urodynamics OR "voiding cystourethrogram" OR "imaging or nuclear medicine" OR "PET scan*" OR "positron emission tomography" OR Fluoroscopy OR "DEXA scan*" OR "dual energy X-ray absorptiometry" OR "EOS scan*" OR "EOS imaging" OR LINAC OR "linear accelerator" OR IVP OR "intravenous pyelogram*") N7 ("child life therap*" OR plaything* OR "play therap*" OR "play specialist*" OR "role play*" OR "clown doctor*" OR "art therap*" OR play OR animation* OR comic OR comics OR "virtual reality" OR "augmented reality" OR "mobile applications" OR DVD OR leaflet* OR cartoon* OR book* OR storybook* OR game* OR website* OR storyboard* OR app OR apps OR ipad* OR "toy scanner*" OR "toy MRI" OR "mock scanner*" OR "mock MRI" OR lego OR photobook* OR mock OR video* OR therapeutic OR mHealth OR "online system*" OR picture* OR internet OR "patient information" OR film* OR "educational intervention" OR psychological)))

Appendix C: Table with detailed data charting and

Author/Year/ Country	Aim	Study Design	Participants age & condition/s	Intervention characteristics	Intervention delivery	Data collection methods	Outcomes	Results/Findings
Ashmore et al (2019) UK	To gain feedback on the initial implementation of the app to help inform further enhancements of the resource	Descriptive quantitative design	23 children (median age 9 years, range 4 to 12 years). who had never had an awake MRI (n=19/23) or had had an MRI more than 1 year ago (n=4/23).	An **app** (targeted at 4–12-year-old children) to produce an immersive 360º VR experience of the entire MRI journey, showing (1) arriving at the MRI reception and the waiting area, (2) participating in the MRI safety screening process, (3) highlighting where the radiographer sits during the scan, (4) introducing the scan room, (5) allowing them to experience being in the scanner, and (6) saying goodbye after their scan.	The resource was developed to be used by health play specialists in hospital (using a Z4 mini headset) but could be used at home where a disposable Google Cardboard version 2 headset was mailed to patients.	Locally developed parent questionnaire<br>Likert scales 0-10<br>How easy was the app/booklet?<br>How enjoyable was the app/booklet?<br>How helpful was the app/booklet?<br><br>Likert scale (5 point, strongly agree to strongly disagree)<br>The app/booklet answered my child's thoughts/questions about having an MRI<br>After using the app/booklet my child feels more positive about having an MRI<br><br>Would you recommend the app/booklet to other young people? (yes/no)<br><br>Locally developed HCP questionnaire.	Ease of use<br>Helpfulness of information,<br>Enjoyability of the resource	23 parent/carers answered the questionnaires.<br>The parent questionnaire highlighted a positive response to the resource.<br>How enjoyable did your child find using the app/booklet? (Median =8.5)<br>How helpful did your child find the app/booklet? (Median =8)<br>How easy to use did your child find the app/booklet? (Median =10)<br>The app/booklet answered my child's thoughts/questions about having an MRI (Agree)<br>After using the app/booklet my child feels more positive about having an MRI (Agree)<br>The feedback showed a positive impact of the app on parents, the app allowed them to better understand their child's upcoming MRI, helping to reduce their own anxieties and enabling them to better prepare their child.<br><br>10 health professionals answered the questionnaire; health play specialists (2/10), radiographers (7/10), and a health care assistant (1/10;<br>staff members believe the preparation resource to be a useful tool.<br><br>Of the 5 patients originally booked for MRI under GA, 4 were able to tolerate an awake MRI
Barnea-Goraly et al (2014)<br><br>USA	To judge the feasibility of using a behavioural desensitization program to yield high quality brain MRI scans in sedation-free children	Descriptive quantitative study	222 children (4–9.9 years), 147 with type 1 diabetes and 75 age-matched non-diabetic controls	**Multi-media resource and mock scanner** to prepare and desensitise children prior to an MRI.<br>Home preparation (a) a video describing the MRI environment and procedure, (b) recordings of MRI sounds played to the child at home and (c) a brochure describing a game designed to encourage the child to practice staying still like a statue ("The	One part delivered by parents at home and one part delivered by staff within the radiology centre.	Each scan taken was reviewed independently by 2 staff to discern if they were useable and of good quality.	Useable scan<br>First attempt successful scan.	Brief behavioural training can lead to a high rate of success for obtaining excellent-quality brain MR images without sedation from very young children.<br>92.3% of the total cohort had usable T1-W scans and 78.4% had usable diffusion-weighted scans after the first attempt.<br>There was no significant difference between scan success rates for children with type 1 diabetes and controls. Two centers used the inexpensive mock scanner for child preparation. The other

				Statue Game"). Preparation at the Hospital - a mock-MRI session. Children underwent practice sessions (30–60 min) in an MRI simulator supported by staff trained to use child-friendly interactions to promote the child's comfort and motivation during the MRI scan.				centre (45 children) had video capabilities and had similar success rates to those observed using a commercial mock scanner. One centre (45 children) did not have video capability during the real MRI scan (an important contributor to scan success)<br>Specifically, there was 100% success for T1-weighted scans using both mock scanners and 93.3% success rate for diffusion-weighted scans using the inexpensive mock scanner compared with 94.7% success rate for centres using the commercial mock scanner (P=0.72). At the centre that used the inexpensive mock scanner without video capabilities the success rates were 91.2% for diffusion-weighted scans and 100% for T1-W scans.
Bharti et al (2016)<br><br>India	To evaluate the effectivenes s of an MRI specific play therapy intervention on the need for sedation in young children	Randomized control design	79 children (40 intervention group, 39 control group) undergoing MRI for neurological and non-neurological conditions. Children's mean age was 7.11 y (SD=2.06)<br><br><br>Children were excluded if they had developmental disorders, mental retardation or had previously undergone a diagnostic MRI.	Children in the intervention group received MRI customized play therapy with a doll sized **mock scanner in** the outpatient department on the day of the MRI investigation.	Play therapy sessions were conducted by a paediatrician and a trained medical social worker on the day of the MRI investigation	The scan quality was rated on a five-point scale by an experienced radiologist (blinded to the intervention received by a child) 1: no motion artifacts, excellent quality, 2: little motion artifacts, good quality, 3: moderate motion artifacts, acceptable quality, 4: excessive motion artifacts, poor quality, and 5: incomplete scan.<br><br>If the child did not cooperate with the procedure within 20 minutes the standard protocol for sedation was used.	Number of children requiring sedation<br>Quality of the scan achieved	Out of 40 subjects in the intervention group, only 20 % (n= 8) required sedation whereas in control subjects, 41 % (n=16) needed sedation during MRI scan ($\chi 2 = 4.13$; P=0.04). This was statistically significant.<br><br>The study supported the effectiveness of MRI customized play therapy with children prior to the scan as it significantly reduced the need for sedation and anaesthesia in a significantly greater proportion of children as compared to the control group.
Capurso et al (2020)<br><br>Italy	To establish and evaluate an MRI preparation procedure	Retrospectiv e cohort study	66 children (3–14 years; mean 7.52 y, SD 2.55 y, 63% male) were prepared	**Play-based stimulation MRI training** using a 8-step protocol included reading a booklet about the MRI, listening to the sounds made by the MRI and comparing the sounds to other known daily	The training protocol is completed by two volunteers	Quality was measured through a 4-point motion artefact scale at 3 points during the MRI<br>1 = no motion artefact and excellent quality; 2 = little	MRI pass rate<br>Quality of the obtained images.	All the children succeeded in completing the preparation. Out of the 66 prepared children, 62 (93.9%) completed the MRI scan, and 4 refused to enter the scanner or became agitated before commencing the scan.

			to undergo MRI scans.	objects, role-play activity where the child performs an MRI scan on a doll and plays the sounds made by the scanner, an indoor obstacle course where a child goes through a cloth-covered play tunnel and then has to stop and lie still for a few minutes while the volunteer plays the sounds.	An average intervention lasted approximately 70 minutes.	motion artefact and good quality; 3 = moderate motion artefact, acceptable quality; and<br>4 = excessive motion artefact, poor quality.		Of the 62 MRI scans, 4 had a mean score > 3 (in the De Bie's scale, a score of 4 indicates poor image quality). Out of 66 children who underwent our MRI preparation, 61 (92.4%) achieved clinically diagnostic scans. 1 1 scan was deemed to be almost useless.
de Bie et al (2010)<br><br>Netherlands	To evaluate the use of a mock scanner training protocol for preparation of children of 3 to 14 years of age for both structural and functional MRI	Descriptive quantitative design	90 children (median age 6.5 years, range 3.7–14.5 years) 47 children (MRI group)<br>43 children who were recruited for a controlled study on brain development, intelligence, and cognitive outcome in children born small for gestational age (fMRI group).	**Full-size mock scanner training**. Children sitting next to the mock scanner whilst receiving verbal instruction on the purpose of the MRI investigation, the importance of keeping still the parts of the MRI unit and each step of the procedure. The various MRI sounds were played at increasing volumes. Younger children (<7) placed their teddy in the scanner during instruction. Children were encouraged to lie down in the mock scanner, equipped with headphones, and immobilized with foam cushions. A training session was rated a pass when a child was able to lie still for 5 min in the mock scanner while the recorded sounds were heard at maximal volume.	A paediatrician or experienced child-life specialist conducted the training session.<br><br>A training session lasted 30–60 min.<br><br>Delivered before the MRI	The quality of structural MRI scans was rated by a five-point rating scale by an experienced radiologist as 1: no motion artifacts, excellent quality, 2: little motion artifacts, good quality, 3: moderate motion artifacts, acceptable quality, 4: excessive motion artifacts, poor quality, and 5: incomplete scan. Scans of score 1– 3 were considered to be of sufficient quality for diagnostic purposes.<br><br>Success rate of structural scan sessions was defined as the proportion of children with structural MRI scans with score 1–3.	Pass rate of the mock scanner training sessions (ability to be still for 5 mins)<br><br>MRI scan quality	The overall pass rate of the mock scanner training sessions was 85/90. Structural scans of diagnostic quality were obtained in 81/90 children, and fMRI scans with sufficient quality for further analysis were obtained in 30/43 of the children.<br><br>Even in children under 7 years of age, who are generally sedated, the success rate of structural scans with diagnostic quality was 53/60. FMRI scans with sufficient quality were obtained in 23/36 of the children in this younger age group.
Carter et al (2010)<br><br>Australia	The aim was to determine whether the introduction of a mock MRI service assisted in reducing the number of GAs being performed on children undergoing MRI.	Retrospective audit	Children aged 3-14 years 11 months who completed an MRI.<br><br>4 groups; (1) Children who underwent an MRI in the pre-mock period. (2) All children who underwent an MRI in the post-	**Graded exposure to the MRI process and to practice** for the MRI in a 1-hour pre-booked session included;<br>1. Assessment of anxiety (child and their caregiver)<br>2. An educational and preparatory procedure was delivered to allow the child to prepare for the MRI at his or her own pace and level (viewing a chosen movie during the mock MRI, hearing the MRI sounds via a stereo system.	Paediatric occupational therapist One hour session	Retrospective audit of the picture archiving communication system (PACS), medical charts and anaesthesia records	The need for GA<br><br>Completion of the mock MRI<br><br>Number of MRI scans performed<br><br>Quality of the MRI	In the pre-mock period a total of 756 children underwent 1,072 scans. In the post-mock period 875 children underwent 1,205 scans. Of this group, 132 children underwent a mock MRI prior to a clinical MRI.<br><br>In the pre-mock period 756 children underwent 1,072 MRIs with a GA rate of 26.8%. In the post-mock period 875 children underwent 1,205 scans with a GA rate of 18.2%. This overall difference of 8.6% was calculated as being statistically significant using Fisher exact test (P<0.05)

			mock period. This group includes some children who had a mock MRI and some who did not (3) Children who underwent MRI in the post-mock period who had a mock MRI (4) Children who underwent a MRI in the post-mock period who did not have a mock MR	A child was deemed by, an occupational therapist, as "passing" the mock MRI if he or she could remain still in the scanner for 7 mins.			scan	The overall rate of GA in the mock group (all age groups) was 21.2% compared to 17.8% in the non-mock group. This did not demonstrate a statistically significant difference (P
Cavarocchi et al (2019)<br><br>Italy	To evaluate the introduction of the Kitten Scanner training protocol on children undergoing an MRI	Retrospective cohort study	Children aged 4-14 years Group 1 includes all children (n = 570) who underwent the MRI examination before the Kitten Scanner introduction and group 2 includes all children (n = 891) who underwent the MRI examination after its introduction	**Play therapy training sessions**. Children were engaged in a simulation of the real MRI investigation with a toy-model scanner called Kitten Scanner. The children were invited to choose a toy-animal ''patient'' and to place it in the model scanner to start the examination. At this point a short animated video played script explaining why the toy-animal ''patient'' needed the MRI examination, what is an MRI scan, and how the examination is performed, using a child-friendly language.	Child life specialist<br><br>Delivered the same day of the MRI in a quiet room in the department.<br><br>The duration session could last between 30 minutes and 40 minutes.	The quality of MRI images taken after the Kitten Scanner training was evaluated by an experienced radiologist.	Number of children undergoing a brain MRI scan without sedation<br><br>Quality of scans	After the introduction of the Kitten Scanner training, there was a significant increase in the number of children undergoing the brain MRI scan without sedation, both for the total group ($p < .001$) as well as for the four to nine years age group ($p < .001$) Children who received most benefit from this training were in the four to nine years age group.<br>All brain MRI examinations performed without sedation after the Kitten Scanner training were of sufficient quality to be used for diagnostic purposes.
Cedja et al (2012)<br><br>USA	To examine the use of the Preparation and Support Procedures (PSP) program and its effect on the ability of young children to	Retrospective review of medical records	71 children with sickle cell disease (SCD) aged 5.6- 12.9 years (median age 9.9 years) who underwent a conventional MRI of the brain or an R2*MRI of the liver	**The play therapy session** used a small model MRI machine, pictures of the MRI suite and recordings of MRI sounds to prepare the child for the procedure. The sequence of events for the MRI exam and sensory information, including sight, sound and touch, was provided using age-appropriate explanations.	Child-life therapist	The quality of images was evaluated by a neuroradiologist or paediatric radiologist	Quality of the scan Use of sedation or anaesthesia	The child life specialist offered PSP to 33 (46.5%) children.<br>There were no differences in gender among children who received PSP and those who did not; however, PSP participants were younger than nonparticipants (median ages 8.9 vs. 10.9 years, P00.0002).<br>Children who underwent PSP had 4.1 (95% CI 1.0, 16.2) times the odds of completing an interpretable MRI exam

	successfully complete brain MRI or liver R2*MRI exams without the use of sedation/anaesthesia			At the end of this session a coping plan was developed tailored to the individual needs of each child and family.				compared to children who did not receive PSP (P=0.0458)<br>After adjusting for age, children receiving PSP had 8.5 (95% CI 1.7, 43.3) times the odds of successfully completing an interpretable MRI exam compared to those who did not receive PSP (P=0.0098). Of the 30 children who successfully underwent MRIs with the PSP intervention, 20 (67%) had required sedation/anaesthesia for a previous MRI.
de Amorim e Silva et al (2006)<br><br>Australia	To evaluate the effectiveness of a practice magnetic resonance unit, in preparing children to undergo magnetic resonance procedures without general anaesthesia (GA) or sedation	Retrospective review of medical notes	134 children undertook a practice MRI (aged 4.1–16.1 years, median age 7.7 years, 47% boys).	**Practice full scale mock MRI** and children are shown a storybook of a child having an actual MRI with photographs. The steps of the procedure, the adults involved and any sensations that are to be expected are described. If necessary, an adult, an older sibling or a soft toy is used to model the procedure.<br>A child is considered to pass the practice MRI if he or she can lie still for 5 min in the tunnel while the appropriate recorded MRI sounds are being played.	Most practice MR sessions take 30 min to an hour	Retrospective review of the records of children who would otherwise have been referred to GA and attended the practice MRI. Each record was assessed as to whether the child had passed or failed the practice MRI intervention. Children who were considered to have passed and proceeded to a clinical non-GA MRI had the report of the clinical scan reviewed. If the scan had been reported as non-diagnostic because of movement artefact it was classified as a failed scan, otherwise it was considered a pass.	Ability to have an MRI without a GA<br>Scan quality	In all, 120/134 (90%) passed the practice session; 117/120 (98%) of those subsequently had a clinical non-GA MRI and 110/117 (94%) passed.<br>No significant difference with respect to age and sex was identified in those who passed or failed both the practice and diagnostic MRI.
Durand et al (2015)<br><br>USA	The aim of this study was to assess the impact of child life evaluation for children undergoing MRI before referral for general anaesthesia	Before and after design	Children aged 5-18 years. Excluded severe neurodevelopmental delay, inability to communicate verbally and extreme claustrophobia.<br><br><u>Baseline (before) group</u><br>Child life evaluations and interventions were performed only when	**Child life specialist preparation and information giving**. Before the date of the examination, the CCLS informed the child and family what to expect on the day of the examination. On the day of the examination, the CCLS met the child and coordinated care over the course of the visit to ensure optimal comfort. Before the examination, the CCLS discussed coping mechanisms with the child, asking what mental techniques.<br>he or she used when coping with everyday stressors and	Child life specialist<br>Day of the MRI scan	For both elective and mandatory referrals, the following data were collected: date of referral; date of evaluation; date of scan; whether the scan was successfully completed; and whether the scan was performed under general anaesthesia, with diazepam, or with no sedation. The following data for all examinations performed during the study period were extracted from the radiology information system: date of examination, age of patient, and whether general	Successful completion of the scan<br>Need for GA<br>Need for sedation	During the baseline period, 564 of 2,433 (23.18%) CCLS-eligible MRI patients underwent general anaesthesia. During the intervention period, the number of age-eligible patients undergoing general anaesthesia dropped to 484 of 2,526 (19.16%). The difference in general anaesthesia use between the time periods was highly statistically significant (P < .001 by z test for difference between proportions), with an absolute reduction of 4.02% and a relative reduction of 14.18%.<br><br>Among children aged 5 to 10 years, the difference in general anaesthesia use between the baseline period (435 of 958 [45.4%]) and the intervention period (368 of 1,040 [35.4%]) was even more

			specifically requested by the referring provider (47 children)<br><u>Intervention group</u><br>All children scheduled for MRI scans of 60 min in duration underwent mandatory child life evaluation before referral for general anaesthesia (263 children)	encouraging the use of guided imagery.		anaesthesia or conscious anxiolysis was used (diazepam at our centre)		significant (P < .001 by z test), with relative and absolute reductions of 15.4% and 10.0%, respectively. By contrast, the trend toward modestly decreased general anaesthesia use in those aged 11 to 18 years did not reach significance (129 of 1,427 vs 116 of 1,486, P ¼ .35).<br>During the baseline period, 47 patients were referred for child life evaluation, all of whom eventually underwent successful scans. Of these patients, 20 required diazepam anxiolysis and 27 required no anxiolysis. Average monthly child life referrals were 3.9, with a high degree of variability (a standard deviation of 1.9 per month or 49% variation). During the intervention period, 263 patients were referred for child life evaluation. Average monthly child life referrals were 21.9, with a low degree of variability (a standard deviation of 4.0 per month or 18% variation). Fifty-two percent of referrals (125 of 263) resulted in MRI without general anaesthesia, and the scan success rate in this population was 98.4%, with 2 failures due to anxiety. Of the 136 successful cases with child life, 99 (73%) required no sedation and 37 (27%) required diazepam.
Fegley (1988)	The purpose of this study was to examine the effects of choice in pre-procedure instruction on a) children's search for information during radiologic procedures; b) children's behavioural responses	Randomised controlled trial	61 children ranging in age from 4-12 years (M = 7.45, SD = 2.62 who were scheduled for a routine intravenous pyelogram (IVP) and/or voiding cystourethrograms (VCUG). There were no significant differences between groups in gender, radiologic procedures,	**Aim:** To provide choice in pre-procedure instruction<br>**Type:** The child was randomly assigned to one of the following groups.<br><u>Contingent Instruction.</u><br>Individualised education based on children's questions and information needs<br><br><u>Noncontingent Instruction.</u><br>Predetermined standard information about the radiologic procedure.<br><br>After the instruction was completed, the child returned to the waiting room.	Nurse<br>Delivered on the day of the radiological procedure.	<u>Observations</u><br>The observations (by two observers, with high interrater reliability and were blind to the child's group) were recorded at three time periods during the procedure.<br>The proportions of observations for looking, listening, questioning, and exploring were added together to form the 'search for information' score. The possible range of scores was 0-100.<br><u>Manifest Upset Scale</u>.<br>A five-point scale designed to reflect the emotional state of a child.		An examination of the partial correlation coefficients revealed that type of instruction was significantly related to one dependent variable, search for information (pr = -0.29) (p c 0.05). The direction of the relationship indicated that the children who received contingent instruction spent a smaller proportion of time searching for information than those who received noncontingent instruction.<br>Older children spent more time searching for information, (pr = 0.28) were more cooperative laying on the table (pr = 0.50) and during the intrusive procedure (pr = 0.45) displayed less upset behaviour getting on the table (pr = 0.40) and reported less distress (pr = -0.40). There were no significant age-by-instruction group interactions.

	during radiologic procedures; and c) children's self-reported distress response to radiologic procedures.		reasons for the radiologic procedures, and previous experience with the radiologic procedures.			<u>Cooperation Scale</u><br>A five-point scale to measure cooperativeness, defined by behaviours which facilitate procedures.<br><u>Self-report of distress.</u><br>A five-item measure where children were asked to rate how upset, sad, mad, and scared they felt during the radiologic procedure. They were also asked to rate on a four point scale how upset they would feel if they had to have another x-ray.		The first interpretation suggests that since contingent instruction was based on the children's questions and requests, it better met the children's information needs. Therefore, the children needed to search for less information during the radiologic procedures.<br>The second interpretation suggests that the choice of information prior to radiologic procedures enhanced feelings of constraint rather than feelings of self-determination.
Fraser (2019)<br><br>USA	To examine the effects of choice of information in pre-procedural instruction on children's responses to select radiologic procedures.	Electronic medical record review	958 children aged 3 and over have participated in the programme over a 6-year period	Patient Awake While Scanned (PAWS) **preparation and support program** which involved phone assessment 2 weeks before MRI. CCLS staff send an email with pictures of MRI camera and tips.<br><br>30 mins before appointment CCLS meets family to provide individualized support<br><br>MRI technologist and CCLS present for scan and if needed patient receives coaching, explanation, and support from the CCLS, MRI technologist, and caregiver during the MRI to promote coping and completion of the scan. Following a completed MRI, the child receives rewards.	Mostly Certified Child Life Specialists (CCLS), but also MRI technologist and caregiver	Not stated	Completion rate<br>Cost savings	A 96% rate of successful scan completion without sedation<br><br>This program has minimized health risks associated with anesthesia use in MRI and lowered the overall cost to families and the institution. There is a cost saving of $241.82 an hour in salaries alone
Gebarski et al 2013<br><br>USA	To assess the efficacy of a cartoon and photograph montage storybook in preparing	Randomised prospective study	100 children (87 girls, 13 boys)<br>Mean age 5.3 years.<br>50 children received the storybook and 50 did not.	A **storybook** with cartoon characters superimposed on photographic backgrounds of the radiology department and fluoroscopy suite. An accompanying stuffed animal was provided to enhance the	Delivered by the parent/carer at home	Parent questionnaire completed after the VCUG to rate:<br>• their child's tolerance of the exam from 1 to 5, worst to best.<br>• a rating of the book and when they read it	Child distress	The association between experiencing the storybook and high-performance scores as rated by the technologist (Table 1, Fig. 2) was statistically significant (P value=0.0092) with an odds ratio of 2.7 (P value=0.0105). At any age, children prepared with the storybook were 2.7 times as likely to score high.

	children for VCUG.			parent–child interaction during reading.<br><br>No explicit instructions were given, leaving the approach, timing and frequency of reading the storybook to the parent or guardian.		• other sources of information used to prepare for the VCUG.<br><br>VCUG technologist (blinded to which children received the storybook) rated each child's tolerance/distress on a scale modified from the Groningen distress scale at 2 points in the procedure (1 = crying and difficult to restrain or combative, 2 = crying and needed restraint, 3 = crying and needed reminding to hold still but some cooperation, 4 = few tears but cooperative, 5 = not scared, no crying, very cooperative.)		Prior experience of VCUG by the child or parent (63 children) resulted in higher tolerance scores than those (34 children) without prior experience (Table 2), but this was not statistically significant (P=0.6996).
Hallowell et al (2008)<br><br>Australia	To determine the effectivenes s of a PMRI service in helping children cope with diagnostic MRI and to reduce the requirement for GA	Clinical prospective audit	291 children (aged 3 years 7 months to 17 years, mean 7.9 years) undergoing an MRI	**Play MRI process**<br>Children viewed a photo story book, discussed the steps and sensations that might be experienced and could ask questions. The child was then shown the PMRI unit and experienced an MRI procedure at his or her own pace. The child was encouraged to make choices such as selecting a DVD to watch. For those children who required intravenous administration of contrast during the scan, this was discussed alongside coping strategies including breathing strategies. Comfort positioning was also developed.<br>A PMRI session was considered successful if the child was able to stay still in the practice MRI unit for at least 5 min	Educational play therapist<br>Session delivered on the day of the MRI	MRI scan results were reviewed by a paediatric radiologist to ascertain whether the scan was diagnostic, diagnostic with some movement artefact, or non-diagnostic. The clinical MRI was considered to be incomplete if the images obtained were degraded by movement or if the scan was aborted because of excessive movement or patient anxiety	MRI scan quality	Of the 291 children who underwent a PMRI, 218 (74.9%) passed, and 227 (78%) went on to clinical MRI without GA. Of these 227 children, 198 (87.2%) had passed a practice MRI, 1 (0.4%) had failed and 28 (12.3%) had been considered borderline. A diagnostic study was achieved in 218 (96%) of the 227 children who underwent a clinical MRI without GA
Han et al (2019)<br><br>Republic of Korea	To evaluate whether virtual reality education for paediatric	Randomised clinical trial	99 children aged 4 to 8 years who underwent chest radiography	**Virtual Reality group**<br>3-minute virtual reality education explaining chest radiography. Delivered 5 mins before the procedure.	The virtual reality intervention was performed in a separated area 5 minutes before entering the	<u>Children's stress and anxiety</u><br>Amended version of an OSBD scale for radiographic procedures by a blinded single evaluator.	Child anxiety and distress.<br><br>Need for parental presence	The number of less distressed children (OSBD score, <5) was significantly higher in the VR group (38 [77.6%]) than in the control group (26 [52.0%]) and the degree of stress and anxiety measured was significantly lower in the VR group than in

	patients before chest radiography could reduce anxiety and distress in children and improve the radiographic process			**Control group**<br>simple verbal instruction	radiography room. Children in the VR group received a 3-minute VR educational presentation regarding the radiologic process with a head-mounted VR display.	Parents' or guardians' satisfaction<br>Self-reported satisfaction on a numerical rating scale.<br><br>Procedural characteristics<br>The time for the radiographic procedure (time from entering the radiography room to the production of a chest radiographic image) and the number of repeated procedures were recorded by a blinded single evaluator. After the chest radiograph was obtained, the radiology technologist scored the level of difficulty of the chest radiographic imaging for each child using a numerical rating scale	Parental satisfaction score<br><br>Procedure time<br><br>Number of repeated images<br><br>Process difficulty score.	the control group (mean [SD] OSBD score, 2.0 [3.7] vs 5.0 [6.1]; mean difference, 3.0 [95% CI, 1.0 to 5.0]).<br><br>The mean (SD) score for parental satisfaction (9.4 [1.4] vs 8.6 [2.0]) was higher in the virtual reality group than in the control group.
Hartman et al (2009)<br><br>USA	The purpose of this study was to assess if preprocedural education with an investigator developed photo diary decreased preprocedural stress and anxiety for school-aged children 7-12 years undergoing MRI.	Randomised controlled trial	50 children (7-12 years old, without intellectual disability) undergoing an MRI<br><br>25 in control group and 25 in education group.<br><br>Children included had not undergone a previous MRI after the age of 5 years	Education group<br>24- page photo diary provided for children to read describing what children can expect (sounds, sensations)	Paper implies the photo booklet was read by families	Data were collected at three points in time,<br>1. immediately after study enrolment<br>2. approximately 20 min later, before the MRI scan. Child subjects completed a stress and anxiety survey and parents completed one survey. After completing surveys, subjects were randomized to control or education group.<br>3. After reviewing the photo diary or receiving standard pre-MRI scan care, children were resurveyed using the same stress and anxiety tools, and parents were surveyed<br>Children's Stress was measured using the self-administered Children's Stress	Child anxiety<br>Child stress<br>Parental anxiety	At baseline, there were no differences in stress and anxiety total or subscale stress and anxiety scores by group; total stress score p Z .88 and mean anxiety score p Z .35. Post education, there were no differences in total stress scores by group (p Z .88) or in total anxiety scores by group (p Z .16; Table 2); however, education group children had higher general anxiety (p Z .04), that was reflected in greater likelihood to worry about things.<br><br>In parents in the education group, there was a nonsignificant trend for perceptions of greater satisfaction with education, less child anxiety, and greater child readiness for the MRI scan.<br><br>The results of this randomized controlled study suggest that a photo diary does not reduce pre-MRI stress and anxiety in school-aged children and does not improve satisfaction with education in parents who accompanied children undergoing an MRI scan

						Symptom Scale. This tool is a 25-item Likert scale with a range of 0 (never stressed) to 3 (stressed most of the time) that measures stress in school-aged children.<br><u>Children’s Anxiety</u> was measured using a self-administered survey consisting of 22 yes or no responses, adapted from the Revised Children’s Manifest Anxiety Scale (RCMAS).<br><br>Parental perception of their child’s readiness for MRI<br>VAS with a 10-cm horizontal line<br>Parental anxiety<br>VAS with a 10-cm horizontal line<br>Parental satisfaction with the education provided to their child.<br>VAS with a 10-cm horizontal line<br>.		
Hogan et al (2018)<br><br>USA	To evaluate the effectiveness of an educational video vs. standard of care in improving relaxation and procedural understanding among paediatric patients undergoing a magnetic resonance imaging	Pilot randomized controlled trial	50 children 6 to 17 years of age undergoing an MRI.<br><br>Half of the children had undergone an MRI previously and nearly half required an intravenous catheter for contrast dye administration. Half of all participants received an MRI of their head and/or brain	<u>Educational group</u><br>7 min MRI **educational video** on a portable electronic device in the MRI procedure waiting area.<br>The video walks viewers through the process of having a MRI procedure and provides basic information on 1) what a MRI actually is and how images are taken; 2) the noises that patients can expect to hear when undergoing a MRI; 3) MRI myths such as the use of radiation; and 4) members of the healthcare team they are likely to meet during the course of their visit.<br><u>Standard care</u><br>MRI staff prepare patients for the MRI (e.g., by asking patients to change into a gown and	Self-administered video in the MRI department	Data collected prior to MRI Scan<br>Children > 7 years were asked to circle their level of relaxation using a 10-point VAS with “0” representing “Not Relaxed at All” and “10” representing “Very Relaxed”.<br><br>Data collected after MRI scan<br>Two questions requested children to 1) rate how well they understood what they were told about the MRI using a 10-point VAS with “0” representing “Did not understand at all” and “10” representing “Understood Everything”, and<br>2) open ended questions asking what children found	Child self-reported relaxation<br><br>Child self-reported knowledge	Pre-MRI, the mean relaxation score was 6.92 out of a total possible of 10 (SD = 3.25) among the standard care group and 6.72 (SD = 2.35) among the intervention group (P = 0.49). Post-MRI, the mean relaxation score was 7.12 (SD = 3.10) among the standard care group and 7.84 (SD = 2.43) among the intervention group (P = 0.54).<br>With regards to patient understanding of the MRI procedure, patients in the intervention group had higher levels of mean understanding scores than those in the standard care group, although this difference was not statistically significant (M = 9.40, SD = 1.35 vs. M = 8.04, SD = 3.10; Mann–Whitney U Test, P = 0.09).<br><br>The educational video was associated with increased relaxation among children, with

	(MRI) procedure.			remove jewellery, and by speaking to patients during the MRI itself and asking patients to hold still).		most helpful about the MRI education		the indication that it may be the most effective among older, adolescent children.<br><br>A total of 26 patients, half from the control group and half from the intervention group responded that the educational video was helpful in increasing their awareness and understanding of the MRI process
Johnson et al (2009)<br><br>USA	To evaluate whether an instructional coloring book used by a parent along with the child would reduce anxiety among paediatric patients about to undergo a radiology imaging test.	Before (control) and after (intervention) trial<br><br>Control and intervention groups were clustered by time.	3 to 10 years old children (mean age 6.1 years) who were scheduled for outpatient CT, fluoroscopic, ultrasound, or nuclear medicine.<br><br>Excluded MRI and brain imaging – as they all have conscious sedation	**An instructional colouring book**, 'Radiology for Kids: Take a Tour with Garfield' included cartoon depictions of equipment and brief explanations of radiology imaging tests as explained by the Garfield character and Odie undergoes the tests.<br><br>Textual information included mention of some details of fluoroscopy exams (including the need to drink barium and the unpleasant taste), ultrasound exams (including the use of topical gel and the associated sensation), CT exams (including the need to hold still), and MRI exams (including the need for a shot in a vein with associated pain, and the need to hold still).	The radiology colouring book was given to parents and patients for review while in the waiting room before their radiology tests.<br><br>Parents and self-directed educational	<u>Parents</u><br>Parental anxiety - Modified Amsterdam Preoperative Anxiety and Information Scale (APAIS). The modification consisted in replacing the terms 'anaesthetic' or 'procedure' with 'my child's radiology imaging test'<br><br>A visual analogue scale (VAS) to measure parental estimation of patient anxiety level just before the imaging test, using anchors of 'no distress' and 'most distress' on a ten-centimetre scale.<br><br>Parents in the intervention group were asked four specific Likert-scale questions related to their perceptions of the utility of the colouring book in this setting.<br><br><u>Children</u><br>Modified Faces Pain Scale-Revised (FPS-R) to estimate patient anxiety levels, using the identical face images as with the FPS-R but replacing scripted descriptors for pain for descriptors of worry and fear in the verbal instructions.	Parent reported child anxiety<br>Child anxiety	Neither parental estimation of patient anxiety (from the VAS) nor patient anxiety score (modified FPS-R from the patient) differed significantly between the control group with no colouring book and the intervention group who reviewed the colouring book.<br><br>There were no statistically significant differences in the demographics or baseline measurements between the two groups.<br><br>The mean modified Faces Pain Scale-Revised (FPS-R) scores, as obtained directly from patients, did not differ significantly between groups ($p = 0.77$; Table 1). Distribution of responses was nearly identical between group subjects, with approximately two-thirds of subjects in each group choosing the least scared face (Table 2).<br><br>The mean visual analogue scale (VAS) values, as obtained from parents in estimation of patient anxiety level, did not differ significantly between groups ($p = 0.30$)<br><br>In response to the statement, 'The Colouring Book helped me better understand the radiology imaging test my child had', 59.9 percent (100/167) of parents indicated a score of 4 or 5. In response to the statement, 'The Colouring Book helped make me less worried about the radiology imaging test my child had', 55.4 percent (92/166) of parents indicated a score of 4 or 5. In response to the statement, 'The Colouring Book helped

								make my child less worried about having the radiology imaging test', 57.2 percent (95/166) of parents indicated a score of 4 or 5. In response to the statement, 'I was pleased to have received the Colouring Book', 92.3 percent (155/168) of parents indicated a score of 4 or 5.
Johnson et al (2014)<br><br>USA	To examine effectivenes s of the social script intervention "Going to Imaging" application (app) on anxiety, challenging behaviours, and procedure duration among children with ASD, and the anxiety of their parent.	Randomized controlled trial feasibility study	32 parents and 32 children (age 0-19 years) in the study with a mean age of 10.3 years (SD = 5.1)<br><br>Children had an ASD diagnosis by parent report<br><br>Children with planned sedation or anaesthesia were excluded because the iPad social script iPad application did not cover the steps of intravenous insertion or sedation.	Four procedure specific apps for MRI, CAT scan, x- ray and nuclear medicine. Each app has 10 screens of photos. The script was based on social script formatting that prepares a child by breaking down a procedure into steps and provides a script of responses.	The experience of the child using the app was estimated to be 5 minutes.<br><br>A researcher delivered the intervention	The study involved three data collection time points on the same day: (1) immediately before the iPad app intervention and (2) immediately after the intervention, and (3) during imaging. A researcher, who was blinded to intervention collected the data during imaging.<br><br><u>Parent Anxiety</u><br>State-Trait Anxiety Inventory for Adults (STAI-S).<br><br><u>Child stress</u><br>HR and BP monitored via a battery-operated wrist cuff provided by the research team.<br><br><u>Child behavior</u><br>Measured with the three subscales of the behavioral observation tool for children with ASD in the health care setting (BOT).	Stress response<br><br>Observable child challenging behaviours<br><br>Procedure duration.	Pre and post intervention mean child HR and systolic BP for both the TAU and app groups are presented in Table 4. The change in mean HR was greater for the intervention group (drop of 4.8 beats/minute) compared to the control group (drop of 2.3 beats/minute).<br><br>Child systolic BP decreased in the intervention group (5.6 mmHg) and rose in the control group (8.7 mmHg). Children in the control group had higher mean number of challenging behaviours in 2 of 3 subscales of the Behaviour observation Tool.<br><br>The imaging procedure's time in the imaging room was less for the intervention group compared to the control only for the time period D2.<br><br>Change in parents state anxiety was greater for the app (drop of 0.6 points) compared to the control group (rise of 2.5 points). There was a small effect size of 0.33.
Karakas et al (2015)<br><br>Turkey	Study aimed to demonstrate whether pre-scan training and orientation affect fMRI compliance of children with ADHD and	Part of a large-scale descriptive quantitative design	77 boys aged 6-12 years - a subsample (53 boys with ADHD and 24 boys in the control group) of the larger study protocol (70 boys with	Children were taken on a tour of the department, shown the MRI scanner and introduced to staff and technicians. They were allowed to watch as another child was being scanned and were given the opportunity to ask questions. They were treated with reinforces such as candy bars.<br><br>Just before the MRI, a standard	Study coordinator.<br><br>Preparation and training were conducted on the day of the scan.	State anxiety scores.	Scan success (acceptable amount of head motion)<br><br>Repetition rates.<br><br>Cancellation	Compliance was not significantly different between ADHD and control groups based on success, failure, and repetition rates of fMRI. Compliance of ADHD patients with extreme levels of anxiety was also not significantly different.

	determine whether this compliance is modified by state anxiety.		ADHD and 38 boys in the control group). Exclusion criteria included neurological and/or psychiatric comorbidities (other than ADHD), the use of psychoactive drugs, and an intelligence quotient outside the normal range.	training session was conducted outside the scanner in a quiet room. Children were individually trained, and practice trials were repeated until the children understood the task.<br><br>Whilst in the MRI room, the child was introduced to the scanner, head coil, headphones, and response pads. When the child was ready and there were no overt signs of distress, scanning started. Structural scans were performed before the functional scans, with at least 45 min between the structural and functional scans.			s due to refusals<br><br>Expression of distress while in the scanner	
Mastro et al (2019)	To evaluate the effectivenes s of an anaesthesia-free patient- and family-centred intervention through an analysis of MRI quality, health-care costs, and operational efficiency as compared with other approaches.	Retrospectiv e review of electronic medical records	500 children aged 3-17 years, who underwent outpatient MRI. 125 children in each of the four groups.<br>• Intervention group (patient and family centred with no anaesthesia.<br>• Comparison group – no anaesthesia standard care.<br>• Comparison group – certified child life specialist preparation. Anaesthesia given.<br>• Comparison group – standard care, anaesthesia given.	Pre MRI preparation session for child which includes a preparation book on iPad (with sounds, pictures, and text) covering all stages of the MRI visit. A medical play session led by the child, allowing the child to express fears, concerns, or comfort level. A preparation session with a mock toy MRI scanner with figures and dolls. Practice of coping techniques such as keeping still, guided imagery, audio music, and movie with MRI goggles. Finally, the CCLS and/or parent provides intervention and support during the actual MRI.	Nurse developed CCLS supported	MRI quality on a 5-point Likert scale<br>Hospital charges<br>Procedural time	Image Quality<br>Hospital Cost<br>Procedural Time	The PFC/NA intervention group was found to have statistically significant lower costs ($p < .0001$) and shorter procedure times ($p < .0001$), and 96.8% of the MRI images were of acceptable or better quality than those of the SC/A and CCLS/A groups.

McGlashan et al (2017)<br><br>UK	To examine whether the animated educational video provides an internet-based tool for MRI preparation that reduces scan-related anxiety in young children undergoing awake MRI. Secondly, we hypothesise that the animated educational video is accessible to a range of children including those with a neurodisability	Prospective cohort study	6.5 to 11.5 years (9.23 ± 1.68).<br>9 children with A-T (neurodisability with movement disorders) and 12 healthy controls with no group differences in sex undergoing a clinical research MRI scan.<br><br>9 children had previous MRI scans (8 from the A-T group and 1 from the HC group).	**An internet-based educational animated video.**<br>The animation used was an updated version from the Szeszak et al. (2016) study and lasts 3 minutes. The animation is about a young girl called Jess who has an MRI scan. Justification for the characters, dialogue and theme of the animation are described previously.	Self-directed.<br><br>Participants were sent an internet link to the animation prior to the MRI scan appointment so they could watch the animation in advance.	Locally developed questionnaire with closed responses (Likert and yes/no) and some qualitative responses<br><u>Children</u><br>Frequency of watching video, who and how watched video<br>Perceptions of the video (like, would they use elements of it during their scan)<br>Pre-scan perceptions (worry, expectations)<br>Post-scan perceptions (whether the animation helped them undergo the scan, whether it helped them feel less nervous etc)<br><br><u>Parents</u><br>Pre-scan questionnaire on whether the animation was viewed, perceived positively by their child, helped prepare their child for the scan).	Understanding of MRI scan<br>Likeability of the animation<br>Usefulness of the animation in preparing the child for the MRI	Of the 21 children, 9 (43%) watched the animation only once and 12 (57%) children watched the animation two to five times. 18 children (86%) watched the animation with family and 3 children (14%) watched the animation alone.<br><br>When asked how much the child liked the animation the total mean score was 16.9 ± 2.3 out of a maximum of 20 (84.5%).<br><br>the children had a good pre-scan understanding of the MRI procedure with a whole group mean of 7.7 ± 1.6 points out of 10 (77%). Pre-scan anxiety for the whole group was low with a mean score of 5.5 ± 1.3 out of 12 (45.8%) (lower scores indicate lower levels of anxiety). Post-scan anxiety was 47.5% or a mean of 11.4 ± 3.8 out of 24. The impact the animation had on preparing the children for the MRI before their scan was rated good with a whole group mean of 8.6 ± 1.8 out of 12 (70.8%). Impact of the animation post-scan was rated good with a mean of 13.8 ± 3.4 out of 20 (69%).<br><br>There was a significant negative relationship between age and impact on preparation rated post-scan ($r = –0.669$, $p = 0.001$), which approached significant pre-scan ($r = –0.427$, $p = 0.053$) indicating the animation had a larger impact on younger children. This age-related impact was reflected in the free text comments. For example, a 10.6-year-old male from the HC group commented, “It was aimed at younger children”. These comments indicate the older children would have liked a more mature version of the animation.<br>Nine children across both groups commented they wanted more realistic and louder noises in the animation and six children wanted a better indication of scanner size.

								Results from the parent/guardian questionnaire showed 100% of parents agreeing that the animated film helped prepare their child for the MRI scan.<br><br>19 of 21 children completed the core MRI research protocol
Morel (2020)<br><br>France	Evaluated the impact of a teddy bear-scale model of a mock MRI scanner on the anxiety experienced by parents and their children during MRI without general anaesthesia	Prospective controlled trial	91 children (46 girls, 45 boys) aged 4 to 16 years (median age 8 years) (standard deviation [SD]=2). who presented to the ambulatory tertiary centre for an MRI scan.<br><br>Children were excluded because of severe cerebral palsy, severe attention deficit hyperactivity disorder or a lack of communication skills	<u>Intervention</u><br>Mock scanner specially designed to look like a toy to the scale of a teddy bear.<br><br><u>Control</u><br>Explanation given by the MRI technologist	MRI technologist<br><br>Duration not stated.	<u>Ambiance of the preparation room</u><br>4-point Likert scale (relaxing, neutral, frightening, scary).<br><br><u>Child Anxiety level</u><br>Visual analogue scale (from 0, completely relaxed, to 100, extremely stressed) at three different moments: in the waiting room, after the preparation and after the exam<br><br>Overall appreciation of the MRI examination was collected at the end of the procedure.	Ambiance of preparation room<br>Child anxiety level	Anxiety levels before the MRI examination were lower in children after the installation of our teddy bear-scale model of a mock MR scanner with a similar time of explanation during the preparation without several training sessions.<br><br>The ambiance of the preparation room was considered by children as significantly more “relaxing” in 50% in the post- mock period vs. 20%.<br><br>the anxiety level estimated by children was significantly lower after the explanations in the post-mock period. a significant difference between anxiety score in the waiting room and after the exam was also observed<br><br>The anxiety score after the exam was significantly lower for boys<br><br>Concerning the score estimated by the parents, the analysis only revealed a main effect of time.<br><br>Children and parents gave free comments: Five people thanked the department for this initiative. They also reported that they understood the MRI device much better. One child explained that he was still afraid of the possibility of having an intravenous injection during the examination.
Nordahl (2016)<br><br>USA	To develop improved and safer methods for obtaining	Cohort study	17 children aged 9- to 13-year-old children with ASD and	**<u>Pre-visit preparation</u>**<br>Structured interview<br>Video Model<br>Set-up of imaging environment<br>Mock scanner room	Behavior analyst, parents, and the research team	Quality assurance procedure to meet the QA threshold.	Scan success rate (acquiring high-quality diffusion-	Acquiring high quality structural MRI scans in severely affected, minimally verbal children with ASD, and comorbid intellectual disability is possible at a high rate of success without the use of sedation

	high-quality images in a broader spectrum of children with ASD.		intellectual impairment	3T MRI suite<br><br>**Mock MRI session**<br>Full-size mock scanner practice: approaching mock MRI bed, sitting down, putting in earplugs, lying down, lowering the head coil, tolerating movement of bed into scanner, tolerating gradient noises, staying still and gradually increasing the amount of time to lie still to 5–10 min.			weighted images).<br><br>Scan quality (quality assurance for acceptable motion).	The success rate in acquiring T1-weighted images that met quality assurance for acceptable motion artifact was 100 %. The success rate for acquiring high-quality diffusion-weighted images was 94 %.<br><br>The number of mock training sessions never exceeded into two visits. All four participants with IQs in the normal range required only one mock visit.
Ong et al (2018)<br><br>Singapore	To assess the effectivenes s of pre-scan videos on the percentage of children requiring general anaesthesia (GA), having repeated magnetic resonance imaging (MRI) sequences due to motion, and having confidence in keeping still for at least 30 minutes during their MRI examination s	Prospective randomised controlled trial.	789 children (mean age 11.6 years).<br><br>The children were randomly assigned into 3 groups:<br>• control,<br>• regular cartoon video<br>• interactive video combined with regular cartoon video groups.	A 2-minute regular cartoon of a potato character undergoing an MRI examination, and an interactive video where a child is able to assist a panda character undergoing an MRI examination with MRI sound included.	Children were shown the videos in a separate waiting area prior to their MRI.	Children were surveyed before and after the videos to assess the self-reported duration that the child believes he/she can lie still for the MRI examination	Need to anaesthetise or repeat the MRI sequence	There was no significant reduction in GA requirement in both intervention groups as compared to the control group. There was a statistically significant 13% reduction in repeated scan requirement (p= 0.005) in the regular cartoon video group as compared to the control group.<br>For the combined videos group, there is a 19.6% reduction in repeated scan requirement (p<0.001)<br>Viewing of videos did not have a significant effect on GA requirement even after adjusting for confounding effects of age, gender and prior MRI experience.<br>The results of this prospective randomised controlled trial suggest that children benefit from the pre-MRI videos, as evidenced by the significant reduction in the requirement for repeated MRI sequences due to motion artefacts and improvement in the confidence of children in staying still for at least 30 minutes
Pressdee et al (1997)	To describe the implementati on of a play	Retrospectiv e description	169 children aged 4-8 undergoing an MRI plus any	**Play therapy and colouring book**<br>All children are sent a colour-in story book with their	Play Specialist	Not stated	Completion of scan	169 children between the ages of 4 and 8 years have been prepared. Only one of these required MR imaging under general anaesthesia because of poor cooperation

	preparation programme.		older children who were perceived as benefitting from preparation	appointment letter. This describes a visit to the MR unit though the eyes of a child. They were encouraged to colour in the pictures and bring it along when they visit the play specialist.<br>The play specialist explains the procedure to the child and parents in age-appropriate language. Photographs of children or a teddy bear undergoing MR<br>A small model of the MR unit, including a sliding table-top and light, has been constructed and is used to familiarize the child with the layout of the unit.<br>A tape recording of the noise produced during the investigation is also listened to<br>Any questions were answered. A visit to the MR unit itself is sometimes used				resulting in a non-diagnostic examination. A total of nearly 800 MR examinations have been performed on children aged 16 years or younger during this period.<br><br>Parents felt that this preparation had been of considerable benefit in decreasing stress and anxiety caused by the examination and have responded positively to it.
Pua et al (2020)	To familiarise children to MRI scanner environment and improve tolerance to loud and repetitive scanner noise.	Descriptive quantitative study.	Twelve children aged 5-18 (monozygotic twins concordant or discordant for ASD)	Parents took part in a brief clinical interview with a psychologist to gather info to develop reinforcement strategies and motivation strategies individually tailored to each child.<br><br>Parents provided with in house MRI familiarisation package comprising:<br>• MRI orientation video, introducing child to locations in hospital and MRI scanner.<br>• Mobile app with interactive games.<br><br>On-site visit – mock MRI training session<br><br>Individual rewards	Psychologist interview<br><br>Parent delivered video and app	Measurements from an accelerometer device<br><br>MRI quality indices	Scan duration<br>Scan completion	Only one participant failed to meet criteria for acceptable levels of head motion and image artefact control
Rothman et al (2016)	To evaluate a program that	Prospective randomized study.	64 children full instruction aged 8 years ± 2	We used two levels of individual instruction:<br><u>Intervention</u>	Health professional	Spielberger state anxiety inventory. Parents were asked	Anxiety<br>Need for anaesthesia	The frequency of anaesthesia was statistically significantly lower in children who received full (27%) as compared to

Isreal	prepares children for MRI, by means of full or partial instruction as an assistive tool in reducing the need for anaesthesia while performing MRI scan, and to determine the effectivenes s of full or partial instruction in reducing anxiety		57 children In partial instruction aged 8 years ± 3	64 children received full interactive instruction that included an instructional booklet, movie and simulator practice <u>Control</u> 57 children received partial instruction that consisted of only the booklet Instruction occurred while the child waited for the scan		to rank 10 questions that referred to current feelings		those who received partial (47%) instruction (P=0.018). <br>The median anxiety level prior to instruction was higher than the median level after instruction for both the partial and full instruction groups (pre-instruction: 25 vs. post-instruction: 21; P=0.003) and (pre-instruction: 23 vs. post-instruction:18; P=0.014), respectively
Szeszak et al (2016) <br>UK	To evaluate an animation in preparing children for an MRI scan	Descriptive quantitative design	23 children (mean age of 7.65, SD.2.01). <br>Exclusion criteria were (1) previous experience of MRI scans (2) history of neurodevelopme ntal disorder (3) poor English language comprehension	Animation The animation lasted 3 mins and follows Jess as she experiences an MRI scan. Throughout the animation Jess talks to the audience directly, allowing her to explain the process of the MRI scan without the need for a narrator. Additional information is delivered in the form of explanations that Sam gives to Jess before and during the MRI scan. The design of each scene in the animation was based on real-life MRI equipment at the particular department.	Self-directed	<u>Questionnaire</u> Children's knowledge of MRI on the following topics: (1) "What to expect", (2) "Checking for metal objects" (3) "Wearing headphones" (4) "Amount of room in the scanner" (5) "Noise of the scanner" (6) "Keeping still" (7) "Length of scan". <br>Anticipated anxiety before and after watching the animation. The anxiety-based section focused on a Likert scale on 2 topics: (1) "Feeling nervous" (2) "Looking forward to scan". <br><u>Interview</u> Explored children's understanding, anxiety and opinions of the animation	Knowledge Child anticipated anxiety Opinions about the animation (usability and retained attention).	There were statistically significant improvements in children's knowledge in 3 of the 7 questions when comparing pre- and post-animation scores ("What to expect"," checking for metal objects" and "keeping still") <br>Questions regarding anticipated anxiety relating to MRI ("anxiety about having a scan" and "looking forward to having a scan") showed significant improvements of +1 in median score (Wilcoxon signed rank test, ($p<0.05$) 100% of participants responded that they liked the way the animation looked, that the people in the animation looked friendly, and that they found it easy to hear what the people were saying. 95.7% of participants reported that they liked the MRI animation overall. 87% of participants reported that they would like to see more animations of this sort for other hospital tests and treatments.

								Pre-animation feelings regarding MRI scans - Fear and anxiety were the two main themes elicited.<br><br>Post-animation feelings regarding MRI scans - The main theme within this section was an overall increase in confidence surrounding MRI scans<br>The study was conducted using healthy participants who did not have an MRI scan following the animation. Therefore, the results surrounding the animation's ability to reduce anxiety were based on anticipated anxiety only.
Thung (2018)	To determine whether the Yale Preoperative Anxiety Scale (mYPAS) obtained before MRI simulation can effectively predict success of MRI without sedation and to explore if mYAS scores improve post MRI simulation training.	Before and after cohort design	80 participants (43 boys and 37 girls). Mean age of 8.5 SD 3 years. Most common conditions (25 for seizures, 17 headaches, 13 ortho)	Simulation based training using a practice MRI scanner Practice MRI scanner	Child life specialist	Scan duration.<br><br>Child anxiety assessed using mYPAS	Need for sedation or anaesthesia for MRI<br><br>Child anxiety	69 from 80 did not require anaesthesia for MRI after simulation.<br><br>Overall study cohort mYPAS scores improved from 31 (+/- 11) to 27 (+/-9).<br><br>11 children (7 girls and 4 boys) unable to complete scan due to nervousness or anxiety (n=8) and inability to lay still (n=3).
Tornqvist et al (2015)<br><br>Sweden	To determine whether children who receive age-adjusted routines can undergo MRI without deep	Cohort design with two groups studied at different time period.	Control group included 36 children who attended scheduled MRI scans for head or head and spine examinations for diagnostic	All children in the intervention group received. (1) A booklet and a storybook sent home to the families, which was to be read together by the parents and child. (2) A 'doll-size' model of an MRI scanner made with an MP3 player with the MRI sound recorded was shown to the child at the day care unit. (3) The	Not documented	Four parts to data collection<br>1. Demographic Information whether the child had deep sedation/anaesthesia or was awake, the diagnosis, the number of sequences, duration of events, and whether or not the examination was aborted.	Number of children who successfully went through MRI without deep sedation or anaesthesia. Image quality	All 36 children in the control group, 30 with deep sedation/anaesthesia and 6 without, had acceptable MRI examinations compared to 30 of the 33 children who underwent MRI without deep sedation/anaesthesia in the intervention group.

	sedation/anaesthesia and achieve scans with adequate image quality.		reasons between February 2008 and March 2009.<br><br>Intervention group included 33 children who attended scheduled MRI scans for head or head and spine examinations for diagnostic reasons and for brain development between May 2009 and June 2010.	child watched a DVD film while undergoing MRI		2. Image quality and motion artifacts were assessed. The number of sequences with acceptable quality was recorded in a protocol.<br>3. The parents recorded their satisfaction/dissatisfaction with the care of the child in a modified questionnaire called Healthcare Satisfaction Module specific for Hematology/Oncology. The questionnaire has 20 questions in 6 dimensions.<br>4. Costs for the examination were calculated from the price list	concerning motion artifacts<br>Parents' satisfaction with the care<br>Scan costs.	Comparison of parent satisfaction showed no significant difference concerning the total score, information, emotional needs, technical skills, inclusion of family, and general satisfaction. There was a significant difference concerning communication, with the intervention group scoring higher.
Train et al (2006)<br><br>UK	The aim of this study was to evaluate a psychological intervention designed to reduce distress in children undergoing 99mTc-DMSA, without compromising the imaging quality.	Retrospective (control group) and prospective (intervention group) cohort study	121 children in total<br><br>81 children in the baseline/control group (mean age of 3.8 years (SD 3.2)<br><br>40 children in the intervention group (mean age 2.9 years, SD - 2.4).<br><br>Both the baseline and intervention samples comprised children who had been referred for their	Intervention group<br>Families were sent a brightly coloured photo-booklet depicting a child having a scan.'. The story book depicted in a series of photographs, a young child actor attending hospital with his mother for a 99mTc-DMSA scan. The photographs were accompanied by simply worded information with procedural as well as sensory information. There was also a letter giving advice on preparing children for medical procedures (bringing some of the child's favourite toys, books or videotapes to hospital and trying using distraction to help their child cope with procedures). The waiting area was enhanced to be more child friendly.	Researcher	<u>Baseline group</u><br>Parental satisfaction survey 0 (''very dissatisfied'') to 5 (''very satisfied'') completed after their child's scan.<br><br>Rates of sedation and procedure failure established from the medical notes.<br><br><u>Intervention group</u><br>Parental satisfaction survey 0 (''very dissatisfied'') to 5 (''very satisfied'') completed after their child's scan.<br><br>Parental anxiety<br>Parents completed the ''trait'' scale of the Spielberger Anxiety Questionnaire and then the ''state'' scale of the Spielberger Anxiety Questionnaire just before the scan.	Child distress<br>Need for sedation<br>Parental anxiety<br>Image quality	Sedation rates were significantly lower in the Intervention group. The rates of failed procedures and use of intravenous sedation were also lower in the Intervention group, although it was not possible to subject these figures to formal statistical comparison, due to their low frequency overall.<br>Satisfaction rates were significantly higher in the Intervention group, as was the ratio of positive to negative comments. However, only 32/81 (40%) of the parents in the Baseline group returned the service satisfaction survey.<br><br>The children's distress scores before the procedure, were lower in the photo-booklet group than in the standard care group (Fig. 2). However, there were no significant differences between the standard and enhanced intervention groups in terms of distress ratings before cannulation.

			first 99mTc-DMSA procedure 6 months after receiving treatment for urinary tract infection.			Child's distress<br>Rated before cannulation by the doctor, using a 100 mm visual analogue scale labelled at one end ''no distress'', and at the other ''extreme distress''.<br><br>The child's distress before the procedure was blind rated by the radiographer using a visual analogue scale as described above.<br><br>The image quality was then blind rated by a consultant radiologist		Parental ''state'' anxiety was not found to be significantly associated with child's distress before the procedure (r - 0.057, p - 0.732).<br><br>The qualitative comments suggest that the provision of additional information about what families should expect on the day, set out in an appealing childcentred way, increased levels of cooperation and satisfaction.
Utama et al (2019)	To investigate whether the use of an interactive educational animated video is non-inferior to showing two videos (regular and interactive) in improving children's cooperativeness during MRI scans.	Prospective, randomized, non-inferiority trial	558 children (aged 3 to 20 years old)<br><br>.	Children were randomised to two groups which received the following:<br><br>Group 1 Children (n=281) watched a 2-minute regular animated video which told the story of a boy undergoing an MRI scan and a 2-3 minute animated interactive video where children help a panda through an MRI scan.<br><br>Group 2 Children (n=277) watched the interactive animated video only.	The videos were watched in the waiting area prior to children attending their MRI scan.<br><br>Videos lasted 2-3 minutes.	Children were asked to assess their confidence in staying still for at least 30 minutes both before and after watching the videos.<br><br>Recorded number of children requiring repeat MRI or GA.	Repeated MRI sequences,<br><br>Need for general anaesthesia (GA)<br><br>Improvement in children's confidence of staying still for at least 30 minutes.	Interactive video group 31% (n=86) needed repeat MRI, 0.7% needed GA and proportion of children who reported confidence to stay still increased by 22.1%<br><br>In the combined video group, 36.3% (n=102) children needed a repeat MRI, 2.1% of children needed a GA and the proportion of children who reported confidence to stay still increased by 23.2%
Waitayawinyu (2016)	To identify the success rate of MRI in 6-15 years old, non-sedative paediatric patients after watching MRI	Prospective interventional study	55 children (aged 6-15 years)<br><br>Children were excluded if they had neurovascular diseases contributing to resting tremor or	An introductory video which was presented as both cartoon animation and real MRI set up, narrating in a kids-friendly tone and age-appropriated context. The contents included scanner suite introduction, how the scanner works, patient's position in scanner and audio of the scanner. Patients would then make decision whether they	5 minutes	Data collection record form<br>1) Patient demographic (age, gender, ASA physical status, comorbidity, mental status, history of radiologic procedures, history of anaesthesia, using of contrast media in the study, procedure time and part of MRI), 2) Radiologic information (study	Scan quality<br>Scan completion<br>Use of anaesthetic and/or sedation	After watching the introductory video, 37 participants (67.2%) decided to proceed with non-sedative option. Ninety-four percent of non-sedated group (35 participants) went through MRI scan course successfully while two cases were unable to complete the scan and requested sedation afterwards.<br>The only factor that related the success of MRI scan without sedation identified in this presented study was previous MRI

	introductory video.		any abnormal/ uncontrolled movement.	needed any sedation for the scan session		area, duration of study and quality of MR imaging)<br>3) Anaesthetic data (anaesthetic technique, duration of anaesthesia, anaesthetic agent used and complication.		experience without sedation. Patients of an older age (11-15 year) were likely to succeed without sedation compared with younger age (6-10 year).
Williams & Greene (2015)<br><br>Australia	To examine the impact of the app on children's anxiety when undergoing medical imaging	Prospective cohort study	50 children in the control group 50 children in the intervention (app) group	An App for radiology procedures which includes three training games: keeping still, holding your breath, and receiving contrast medium.<br>To complement the games, there are explanatory videos that link the games with the reality of imaging in the hospital. There is also information for families including tips, things to practice, wearing the right clothes and frequently asked questions.	Children can access the app in a number of ways; Before coming to hospital or when at hospital through the Play Therapists using the app on tablets in the Medical Imaging Department	No information on the data collected	Anxiety<br>Compliance<br>Time taken to be ready for imaging	The average time taken for patients to be ready for imaging reduced from 18.09 to 12.15 min (this included preparation time and/or time spent encouraging the patient to lie down due to reluctance). The average compliance issues reduced from 0.7 to 0.4 (scale 0–4, 0 being no issues) The average anxiety rates (as measured by parents and radiographers) over 4 points (scale 0–4, 0 being no issues) improved as per below<br>Additionally, two patients in the group who did not have the app failed to undergo imaging, while all patients who had the app were able to undergo successful imaging.
Yamada et al (2020)<br><br>Japan	To explore the generalizability of preparation for functional paediatric neuroimaging to clinical simulation in nursing	Retrospective review	241 children aged 4-17 years	A simulation protocol using a mock scanner preparation immediately before an MRI being performed.<br><br>The mock scanner comprises the outer body and scanner bed of an actual MRI. A pair of speakers were installed in- side the scanner so that participants were able to experience the scanning sounds and background noise they would hear during a real scanning. The system was placed in a discrete preparation room	Experienced staff trained in child neurology and developmental and behavioural paediatrics.<br><br>The average simulation time was approximately 40 to 60 minutes.<br><br>Completed just before their scheduled MRI studies.	Medical case note review	Scan completion	Studies were successfully completed for 100 (98.0%) participants with TD and for 130 (93.5%) participants with NDDs, resulting in acceptable-quality MRI.<br><br>school age child subgroups in both the TD and NDD groups showed particularly high success rates<br><br>Regarding age, significant differences in Zero-T application rates were observed between the TD and NDD groups.<br><br>The study suggests, this device can help participants become more relaxed
